# Differences in the Selection Bottleneck between Modes of Sexual Transmission Influence the Genetic Composition of the HIV-1 Founder Virus

**DOI:** 10.1371/journal.ppat.1005619

**Published:** 2016-05-10

**Authors:** Damien C. Tully, Colin B. Ogilvie, Rebecca E. Batorsky, David J. Bean, Karen A. Power, Musie Ghebremichael, Hunter E. Bedard, Adrianne D. Gladden, Aaron M. Seese, Molly A. Amero, Kimberly Lane, Graham McGrath, Suzane B. Bazner, Jake Tinsley, Niall J. Lennon, Matthew R. Henn, Zabrina L. Brumme, Philip J. Norris, Eric S. Rosenberg, Kenneth H. Mayer, Heiko Jessen, Sergei L. Kosakovsky Pond, Bruce D. Walker, Marcus Altfeld, Jonathan M. Carlson, Todd M. Allen

**Affiliations:** 1 Ragon Institute of MGH, MIT and Harvard, Cambridge, Massachusetts, United States of America; 2 Division of Infectious Disease, Massachusetts General Hospital, Boston, Massachusetts, United States of America; 3 The Fenway Institute, Fenway Health, Boston, Massachusetts, United States of America; 4 Broad Institute of MIT and Harvard, Cambridge, Massachusetts, United States of America; 5 Faculty of Health Sciences, Simon Fraser University, Vancouver, British Columbia, Canada; 6 Blood Systems Research Institute, San Francisco, California, United States of America; 7 HIV Clinic Praxis, Jessen, Berlin, Germany; 8 Department of Medicine, University of California San Diego, La Jolla, California, United States of America; 9 Howard Hughes Medical Institute, Chevy Chase, Maryland, United States of America; 10 Heinrich-Pette-Institut, Hamburg, Germany; 11 Microsoft Research, Redmond, Washington, United States of America; University of North Carolina at Chapel Hill, UNITED STATES

## Abstract

Due to the stringent population bottleneck that occurs during sexual HIV-1 transmission, systemic infection is typically established by a limited number of founder viruses. Elucidation of the precise forces influencing the selection of founder viruses may reveal key vulnerabilities that could aid in the development of a vaccine or other clinical interventions. Here, we utilize deep sequencing data and apply a genetic distance-based method to investigate whether the mode of sexual transmission shapes the nascent founder viral genome. Analysis of 74 acute and early HIV-1 infected subjects revealed that 83% of men who have sex with men (MSM) exhibit a single founder virus, levels similar to those previously observed in heterosexual (HSX) transmission. In a metadata analysis of a total of 354 subjects, including HSX, MSM and injecting drug users (IDU), we also observed no significant differences in the frequency of single founder virus infections between HSX and MSM transmissions. However, comparison of HIV-1 envelope sequences revealed that HSX founder viruses exhibited a greater number of codon sites under positive selection, as well as stronger transmission indices possibly reflective of higher fitness variants. Moreover, specific genetic “signatures” within MSM and HSX founder viruses were identified, with single polymorphisms within gp41 enriched among HSX viruses while more complex patterns, including clustered polymorphisms surrounding the CD4 binding site, were enriched in MSM viruses. While our findings do not support an influence of the mode of sexual transmission on the number of founder viruses, they do demonstrate that there are marked differences in the selection bottleneck that can significantly shape their genetic composition. This study illustrates the complex dynamics of the transmission bottleneck and reveals that distinct genetic bottleneck processes exist dependent upon the mode of HIV-1 transmission.

## Author Summary

While the global spread of HIV-1 has been fueled by sexual transmission the genetic determinants underlying the transmission bottleneck remains poorly understood. Here we characterized founder virus population diversity from next generation sequencing data in a cohort of 74 acute and early HIV-1 infected individuals. We observe that the risk of multi-variant infection in men-who-have-sex-with-men (MSM) is not greater than that observed for heterosexuals (HSX), contrary to reports of higher rates of multiple founder virus infections in higher-risk MSM transmissions. These findings were further supported through a metadata analysis of 354 acute and early HIV-1 subjects. We did, however, observe differences between HSM and MSM founder viruses, including a higher selection barrier in HSX transmission with founder viruses being more cohort consensus-like that may be reflective of increased replicative fitness. We also identified a number of residues within Envelope that behave in a risk-dependent manner and could be key for HIV-1 transmission. These novel insights improve our understanding of the HIV-1 transmission bottleneck and underscore the differential selective pressures that founder viruses within the two major transmission risk groups are subjected to.

## Introduction

The global spread of HIV-1 has been fueled predominantly by HSX transmission, with MSM representing a second major risk group [1]. As was first established over two decades ago, HIV-1 undergoes a severe population bottleneck upon transmission with only a limited number of variants from the diverse pool of strains in the source establishing productive infection in the recipient individual [2–6]. While the biological mechanisms underlying this genetic bottleneck remain poorly understood, recent studies support a combination of host factors, including the effective physical barrier of the mucosa [7], availability of target cells [8] and levels of immune activation and genital inflammation that may enhance HIV-1 transmission [9–11]. Recently, in a cohort of transmission pairs it was also demonstrated that factors associated with an increased risk of HSX transmission can mitigate this process and reduce the strength of selection of transmission [12]. The application of single-genome amplification and sequencing (SGA/S) to subjects sampled during acute and early infection has allowed for the inference of the founder virus [13, 14]. In 80% of HSX transmissions a single founder virus is responsible for productive clinical infection [7, 14–18], whereas among MSM the incidence of multi-variant transmission is reported to be higher with up to 40% of infections established by multiple viral variants [14, 19]. These data, coupled with epidemiological data illustrating differential risks of infection based on the route of exposure [20], suggest that the mode of transmission additionally influences the selection of the viral variant(s) establishing systemic infection.

A number of genetic, immunologic and phenotypic signatures of founder viruses, predominantly located within the envelope glycoprotein (Env), have been identified that affect HIV-1 entry [16, 21–28]. In particular HIV-1 clade C founder viruses appear to favor shorter, less glycosylated Envs [7, 21] that are closer to ancestral sequences than their contemporary chronic Env counterparts [29, 30], though this does not appear to be the case in subtype B [31–33]. Korber and colleagues extended these earlier findings and identified a number of Env signature sites enriched upon subtype B transmission [34], many of which may affect Env expression resulting in higher Env incorporation within the budding HIV-1 virion [35]. Aside from these genetic features no dominant phenotypic correlate has been associated with viral transmission other than the preference for CCR5 and CD4+ T cell tropism [4, 14, 25, 36, 37]. Recent studies have shown that transmitted viruses appear to be more resistant to inhibition by interferon-α (IFN-α) than viruses derived from chronic infection [27, 38]. However, it remains unclear whether IFN-α makes a major contribution to the HIV-1 transmission bottleneck as not all studies have observed this property in founder viruses [39, 40].

To date, attributes particular to HIV-1 founder viruses have been identified by comparing these sequences to larger datasets of contemporaneous chronic viruses [23, 25–27], with smaller studies examining epidemiologically linked transmission pairs [7, 21, 41–46]. It is unclear, however, what genetic constraints the mode of sexual transmission imposes on the nascent founder virus. Importantly no studies have directly compared HIV-1 founder viruses specific to MSM versus HSX infections. In this study we used whole-genome amplification and 454 deep sequencing to characterize HIV-1 intra-host genetic diversity in acutely infected subjects and demonstrate, using a genetic distance-based approach, that contrary to previous findings [14, 19], only a single founder virus is detectable in the majority of MSM infections. Moreover comparative analyses of MSM and HSX founder viruses suggests that the mode of transmission imposes differential selection pressures, with HSX viruses experiencing broader, modest selection while MSM viruses exhibit stronger selection but at fewer sites. Furthermore, we identify a number of sites within Env that are differentially enriched upon MSM and/or HSX transmission, suggesting that HIV-1 founder viruses specifically evolve or are selected to overcome the different mucosal barriers imposed by the route of transmission. This study provides important insights into how the mode of transmission shapes the HIV-1 founder virus, as well as into the differing selective pressures between MSM and HSX infection, and may facilitate the design of a more effective HIV-1 vaccine or other therapeutic and prevention strategies.

## Results

### Application of a Hamming distance approach to discriminate between homogeneous and heterogeneous HIV-1 founder virus infections

**Fig 1:**
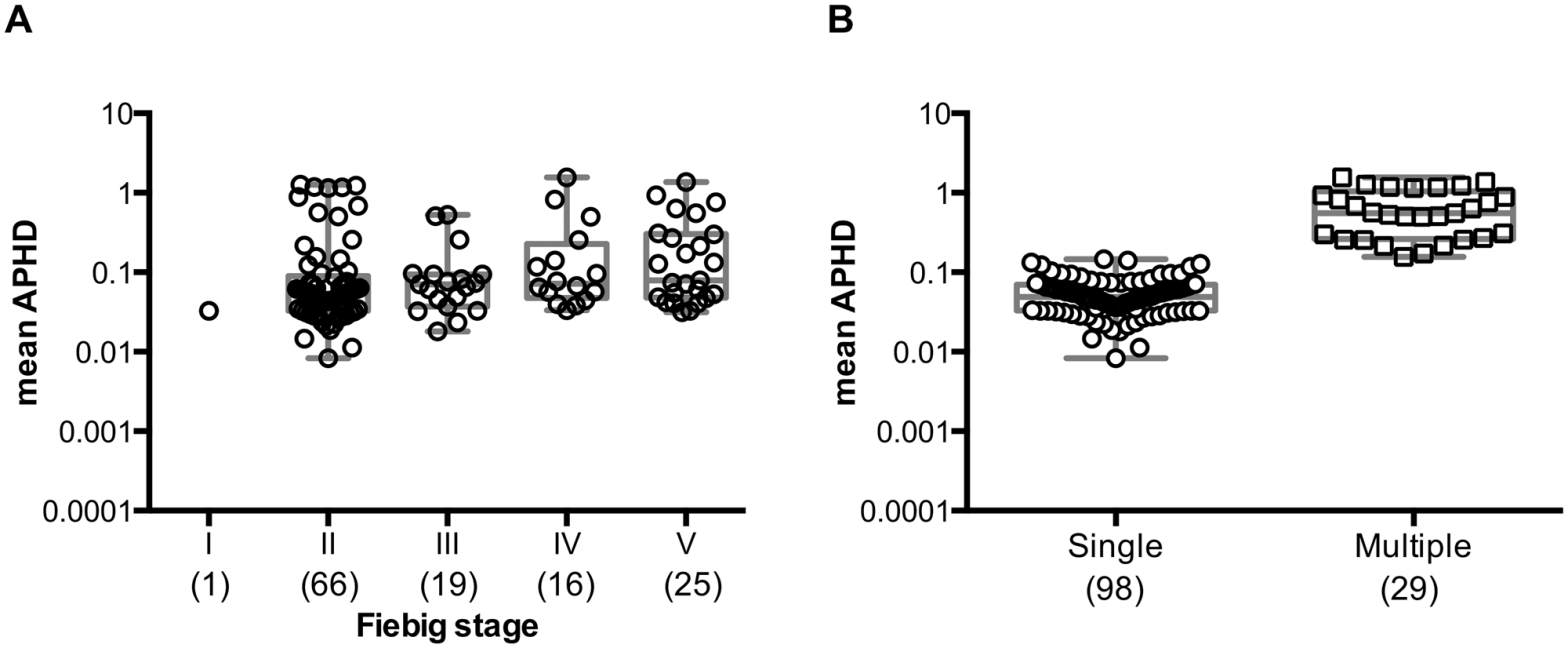
Mean average pairwise Hamming distance (APHD) of HIV-1 Env SGA/S sequences distinguishes between single and multiple founder viruses. **(A)** A training set of SGA/S Env sequences derived from 127 previously published acute HIV-1 infected subjects illustrating a wide range of *env* diversity. The APHD is calculated using a sliding window of 120bp with a step size of 21bp. The mean APHD is plotted according to Fiebig stages as defined by HIV-1 clinical laboratory test results. **(B)** A classifier based on a logistic regression segregated 127 subjects into single or multiple infections and correctly assigned 97% of subjects into the respective groups. Each point corresponds to an individual subject with the number of subjects denoted on the x-axis in parenthesis under each Fiebig stage.

To adapt the application of more sensitive and higher-throughput next-generation deep sequencing data, where the shorter sequencing reads makes the inference of haplotype reconstruction difficult, we implemented a Hamming distance-based genetic approach, termed here the average (over read pairs) pairwise hamming distance (APHD). This method calculates the number of mismatches between reads in a sliding window of a defined size to analyze the level of genetic diversity within the viral quasispecies. To validate this approach we applied it to previously published SGA/S-derived *env* founder virus sequences from 127 subjects (Dataset 1) for which the multiplicity of infection had previously been determined [14, 19] (Fig 1A and Supplementary Materials and Methods in S1 Text). A classifier based on a logistic regression clearly segregated the subjects into those previously identified to have exhibited infection by a single virus (n = 98) versus those infected by two or more viruses (n = 29; Fig 1B), with the model correctly classifying 97% of the subjects. Cross-validated prediction errors and area under the receiver operating characteristic curve (AUC) were used to assess model performance on data not used to build the model. The prediction error based on 10-fold cross-validated AUC was estimated to be 3.95% and the 10-fold cross-validated AUC was shown to be approximately 0.993 with the corresponding 95% confidence intervals [0.981, 1.00]. For each subject, ART_454 software was used to simulate *in silico* sequencing reads with multiple replicates generated resulting in a dataset of 7.9 million *env* reads. Calculation of the APHD score for each subject from each replicate demonstrated a lack of significant difference between the simulated data and actual data suggesting that modeling sample variation and incorporating the error profiles associated with 454 sequencing has not had an undue effect on the classification of subjects. We conclude, therefore, that the APHD approach is suitable for discriminating between homogeneous and heterogeneous infections, and represents an alternative approach to discriminate between single and multiple founder viruses.

### Validation of the APHD approach to deep sequencing data

**Fig 2:**
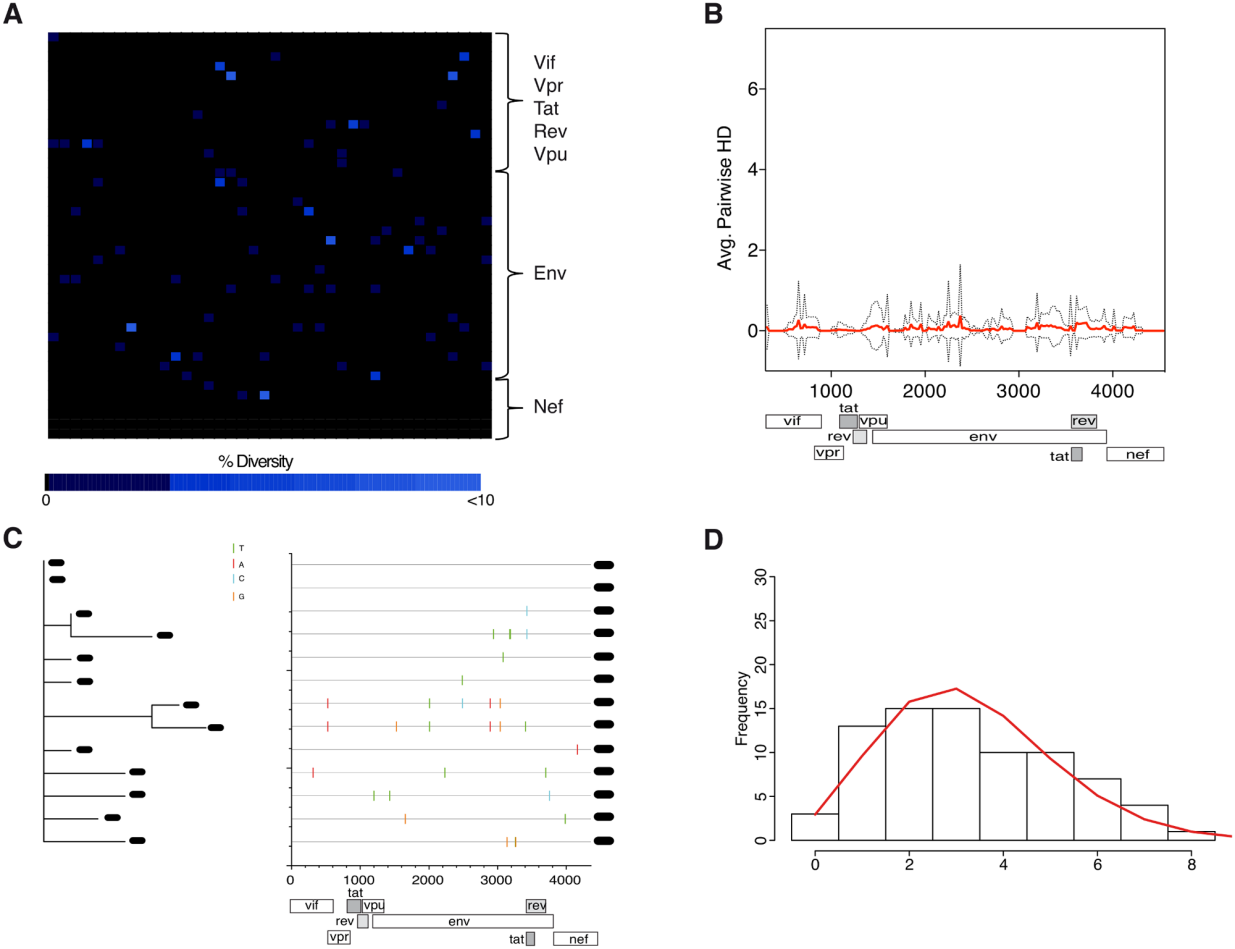
Subject 571373 exhibits low *env* diversity by both 454 and SGA/S reflective of a single founder virus. 454 and SGA/S analysis of 3′ half sequences from subject 571373 (Fiebig stage II/III). **(A)** Heat map illustrating a small number of sites exhibiting low-level amino acid sequence diversity across the 3′ half of the HIV-1 genome as detected by 454 deep sequencing. Plotted is the percentage of amino acid diversity at each position with the first amino acid of Vif located in the top left corner of the grid and last amino acid of Nef located in the bottom right corner. Completely conserved residues are black and low-level variant residues (<10%) are dark blue. **(B)** The average pairwise hamming distance calculated from 454 sequencing reads for the 3′ half of the genome is plotted with the APHD of 0.063 (red line) and standard deviation (dotted black line) shown. The plot shows a relatively uniform population with random sites throughout the genome exhibiting low-level diversity. **(C)** SGA/Ss from subject 571373 covering the 3′ half of the HIV-1 genome display limited structure on a neighbor-joining (NJ) phylogenetic tree (left) and few nucleotide changes from the intrasubject consensus. The highlighter plots (right) compare sequences for each subject’s sequence set to an intrasubject consensus (uppermost sequence) and illustrates the pattern of nucleotide base mutations within sequences using short color-coded bars. **(D)** Hamming distance analysis of SGA/Ss showing infection by a single virus with Hamming distance frequencies conforming precisely to model predictions of a single virus infection (red line). As further support for a single founder virus the estimated time to a single most recent common ancestor (MRCA) of 36 days (23–49 days) overlapped with the estimated clinical duration of infection based on Fiebig stages (18–37 days).

To validate application of the APHD approach for deep sequencing data we performed both SGA/S and Roche 454 pyrosequencing of *env* (see Methods and S1 Text) in 6 individuals from a range of Fiebig stages (II/III to V). In subject 571373 (Fiebig stage II/III) application of the APHD approach to the 454 sequencing data suggested infection by a single founder virus. A codon-diversity heat map illustrates only minor variation (<10%) present in the viral population (Fig 2A), with a low calculated mean APHD of 0.063 (Fig 2B) placing it inside the 75% percentile of APHD scores obtained for the single founder virus group as calculated above. Comparison of over a dozen SGA/S sequences supported infection by a single founder virus with each lineage containing a unique set of near identical sequences (Fig 2C) in agreement with a star-like phylogeny and Poisson distributed hamming distance that conform to a mathematical model of random evolution (Fig 2D).

**Fig 3:**
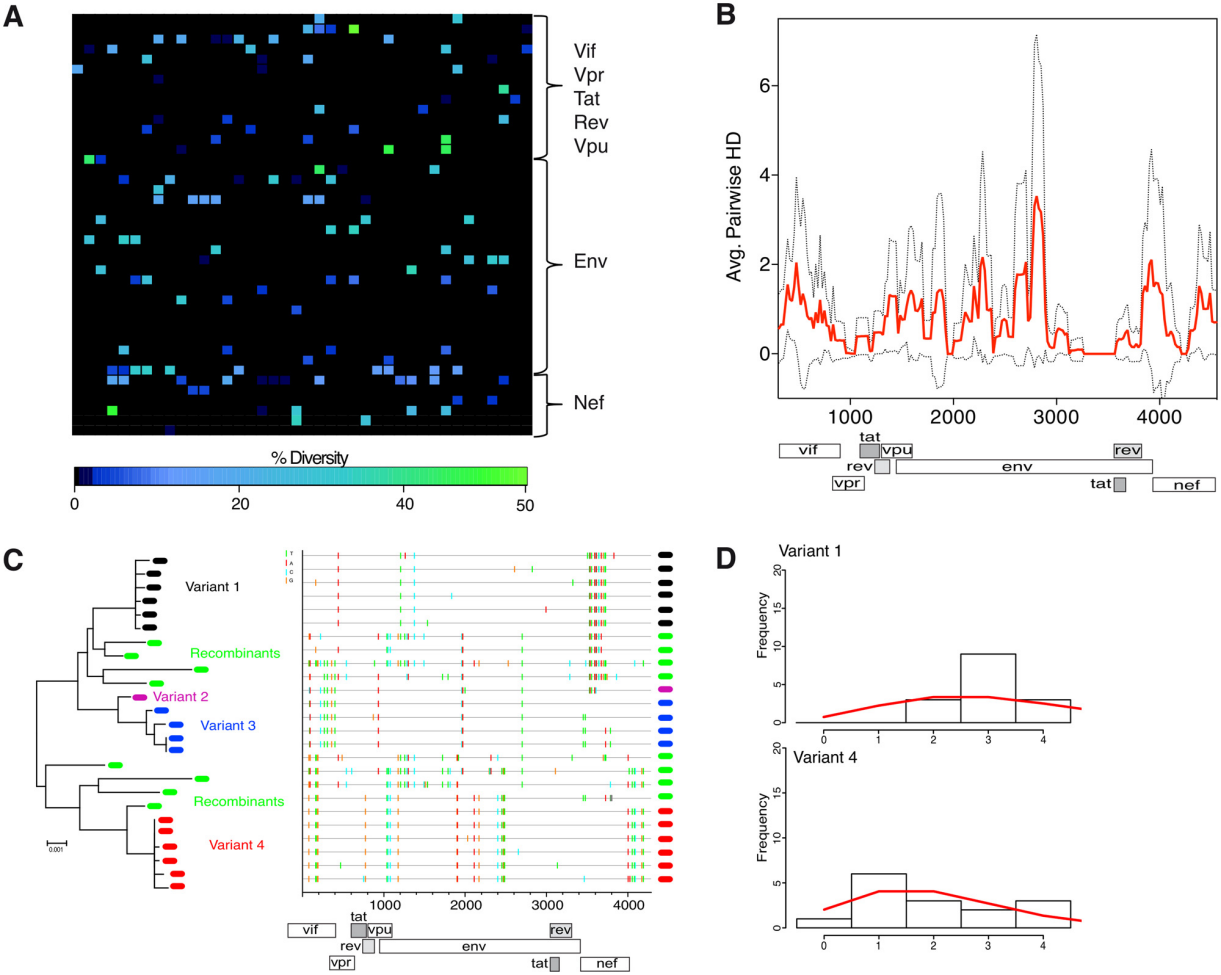
Subject 654207 exhibits high *env* diversity by both 454 and SGA/S reflective of multiple founder viruses. **(A)** Heat maps illustrating a number of sites exhibiting amino acid sequence diversity across the 3′ half of the genome as detected by 454 deep sequencing. Plotted is the percentage of amino acid diversity at each position with the first amino acid of Vif located in the top left corner of the grid and last amino acid of Nef located in the bottom right corner. Completely conserved residues are black and low-level variant residues (<10%) are dark blue, moderately variable residues (20%) are *sky blue* and highly variant residues (>40%) are green. **(B)** The average pairwise hamming distance calculated from 454 sequencing reads for the 3′ half of the genome is plotted with the APHD of 0.752 (red line) and standard deviation (dotted black line) shown. The plot shows a variable population with a high number of sites exhibiting throughout the genome exhibiting high-level diversity. **(C)** SGA/Ss from subject 654207 covering the 3′ half of the HIV-1 genome display a phylogeny (left) revealing productive infection by at least four viruses with inter-lineage recombination. Founder virus lineages are color-coded and labeled variant 1–4. Recombinant sequences are shown by green symbols. The highlighter plots (right) compare sequences for each subject’s sequence set to an intrasubject consensus (uppermost sequence) and illustrates the pattern of nucleotide base mutations within sequences using short color-coded bars. **(D)** Hamming distance analysis of SGA/Ss showing infection by multiple viruses with Hamming distance frequencies (mean Hamming distance of 35.38) not conforming to model predictions of a single virus infection. Splitting of variants into their respective sub-lineages such as variants 1 and 4 demonstrate Hamming distance frequencies that do conform to model predictions of a single virus infection (red line). Subject 654207 is viral RNA positive but Western blot negative (stage II/III of infection).

In contrast, subject 654207 demonstrated a high level of diversity inconsistent with a single virus transmission (Fig 3A), resulting in a mean APHD score of 0.752 (Fig 3B). SGA/S supported infection by at least 4 founder lineages along with extensive interlineage recombination (Fig 3C) while a mathematical model of evolution demonstrated that the SGA/S did not conform to a Poisson distribution (mean Hamming distance per base of 0.005). Notably, however, splitting of variants into their respective sub-lineages did demonstrate conformity to the Poisson distribution and star-like phylogeny resulting in most common recent ancestors (MRCAs) in agreement with clinical estimates (Fig 3D). In next three subjects 882283 (S1 Fig), 702865 (S2 Fig), and 574194 (S3 Fig) the APHD approach again suggested infection by three or more distantly related viruses with high APHD scores of 0.267, 0.680 and 1.580 that was confirmed by SGA/S (which also indicated interlineage recombination). Finally, in subject 1051, deep sequencing demonstrated a high APHD score (0.490) reflective of infection by multiple viruses while our SGA/S data supported at least three founder viruses (S4 Fig). Coincidentally, this subject was also previously examined in detail by SGA/S [14] where at least 4 founder viruses were found (S4 Fig). Therefore, application of our APHD approach to 454 deep sequencing data successfully distinguished between single and multiple founder viruses as validated by parallel SGA/S.

### Majority of MSM HIV-1 infections are established by a single founder virus

To further explore the use of deep sequencing data to examine the multiplicity of infection during the acute phase we next assembled a broader cohort of 74 subjects who had recently acquired HIV-1 either through sexual contact via MSM (n = 64) or HSX (n = 2) exposure including source plasma donors (SPD) (n = 6), or through percutaneous exposure (n = 1) or injecting drug use (IDU, n = 1), (see S1 Text and S1 Table). To reduce the potential of cohort-induced bias, subjects were predominantly selected from two distinct HIV-1 acute cohorts in Massachusetts and Germany. The majority of subjects (88%) were captured very early after infection, with 12 subjects in Fiebig stage I, 53 in Fiebig stage II/III, 5 in Fiebig stage IV and 4 in Fiebig V. All but two subjects were infected with HIV-1 subtype B, and all subjects exhibited high viral loads typical of acute infection with a median of 923,000 copies/ml (IQR: 270,378–4,100,000) and a median CD4+ T cell count of 413 cells/ml (IQR: 310–552).

**Fig 4:**
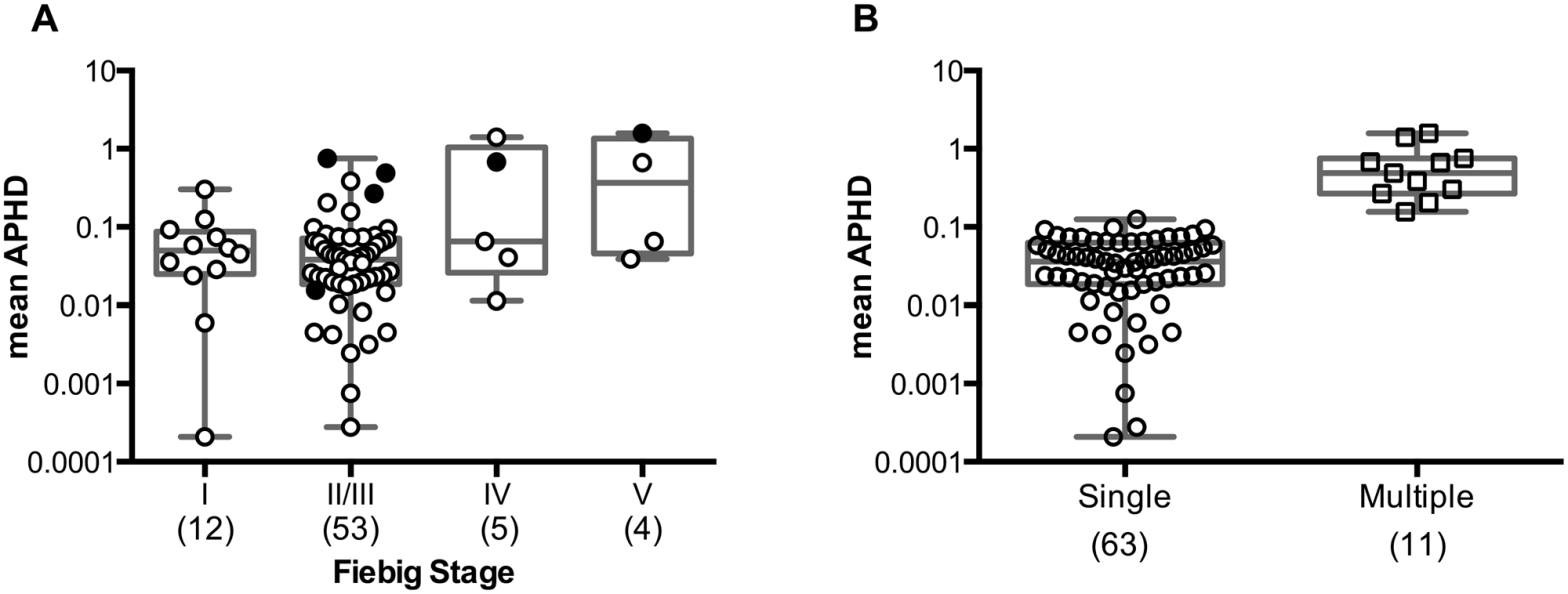
Complexity of acute HIV-1 infection revealed using deep sequencing data and the APHD approach. **(A)** Mean APHD of 74 newly deep sequenced acute HIV-1 infected subjects, illustrating a wide range of *env* diversity plotted according to Fiebig stages. Black circles depict the 6 samples in which SGA/S was also performed. **(B)** Classification of the 74 subjects into single vs. multiple founder viruses resulted in 63 subjects exhibiting a more homogeneous infection suggestive of productive clinical infection originating from a single virus, and 11 subjects exhibiting distinctly higher diversity indicative of heterogeneous infection and infection by multiple founder viruses. Each point corresponds to an individual subject with the number of subjects denoted on the x-axis in parenthesis under each Fiebig stage.

To provide a more comprehensive and informative genome-wide analysis of multiple founder viruses, we PCR amplified the entire protein-coding region of HIV-1 in conjunction with 454 deep sequencing. While sequence coverage varied between samples and amplicons there was no significant difference in sequence coverage between amplicons with a median sequencing depth of 501 for *gag* (IQR: 285–794), 402 for *pol* (IQR: 203–679) and 600 for the 3′ genomic half (IQR: 359–862). Only 4 subjects had a 3’ genomic half sequencing depth < 200-fold with the remainder of subjects harboring sufficient sequence coverage to detect variants at 1%. Utilizing *env* sequences the APHD scores derived from the 74 acute HIV-1 infected subjects ranged from 0.0002 to 1.580 across different Fiebig stages (Fig 4A). The logistic classifier assigned 63 of these individuals to the single founder virus class, with the remaining 11 subjects categorized as infected by multiple variants (Fig 4B). Notably, 6 subjects were previously examined by Keele and colleagues [14] and our approach correctly assigned 5 of these as founded by a single genetic lineage and 1 by multiple lineages. Thus, among the 74 subjects studied the majority (85%) displayed low *env* diversity consistent with single variant transmission. Moreover, given that our cohort was predominantly MSM, this data indicates that the majority of these MSM infections (83%) were established by a single founder virus.

### Impact the mode of transmission has upon the multiplicity of HIV-1 infection

Given that our findings were contrary to the prevailing understanding that higher risk infections such as MSM transmission frequently exhibit multiple founder viruses and that the results of such studies exploring the multiplicity of infection in HSX, MSM and IDU vary widely (Table 1) [7, 14, 15, 19, 44, 47–49], we undertook a meta-analysis of the multiplicity of HIV-1 transmission from a number of studies. We limited our analysis to the 354 subjects (Dataset 2) for whom full-length envelope sequences had been generated, encompassing MSM, HSX and IDU transmissions (the set included the 74 subjects sequenced during this study) [7, 14, 15, 19, 34, 44, 47, 49, 50]. Using the previously determined infection outcome (single or multiple) from these studies, we found the frequencies of multi-variant transmissions were comparable between HSX, MSM and IDU transmission (*P* = 0.167, Chi-Square test; Table 1). Recognizing that 6 of these subjects were counted twice between Keele and our study a re-analysis counting these subjects once still demonstrated no significant difference (*P* = 0.177, Chi-Square test). Taken together, these findings compiled from a number of different studies indicate that at the time of sampling there are no detectable differences in terms of multiplicity of infection between different modes of transmission.

**Table 1:** Multiplicity of HIV-1 infection in HSX, MSM and IDU subjects.

Route of transmission	Study	Virus subtype	Total no. of subjects	Single variant transmission^a^		Multiple variant transmission^a^	
				n	%	n	%
HSX	Tully	B	8	7	87.5%	1	12.5%
	Abrahams [15]	C	69	54	78.3%	15	21.7%
	Haaland [7]	A or C	27	22	81.5%	5	18.5%
	Keele [14]	B	79	65	82.3%	14	17.7%
	Gnanakaran [34]	B	7	4	57.1%	3	42.9%
	***Total***		**190**	**152**	**80.0%**	**38**	**20.0%**
MSM	Tully	B	64	53	82.8%	11	17.2%
	Keele [14]	B	22	13	59.1%	9	40.9%
	Li [19]	B	28	18	64.3%	10	35.7%
	Herbeck [44]	B	9	8	88.9%	1	11.1%
	Gnanakaran [34]	B	9	7	77.8%	2	22.2%
	***Total***		**132**	**99**	**75.0%**	**33**	**25.0%**
IDU	Tully	B	1	1	100.0%	0	0.0%
	Keele [14]	B	1	0	0.0%	1	100.0%
	Bar [49]	B	10	4	40.0%	6	60.0%
	Masharsky [47]	A	13	9	69.2%	4	30.8%
	Dukhovlinova [50]	A	7	7	100.0%	0	0.0%
	***Total***		**32**	**21**	**65.6%**	**11**	**34.4%**

^a^ Chi-Square test was used to examine the association between mode of transmission and infection outcome (*p* value = 0.167)

### Selection pressures are different between HSX and MSM transmissions

Given that we observed no differences in the number of founder viruses between MSM and HSX infection we sought to investigate whether other differences in the founder viruses may exist between the two modes of transmission. To do so we assembled a collection of 131 founder viruses (Dataset 3) derived from acute clade B HIV-1 infected subjects with a defined HSX (55 subjects) or MSM (76 subjects) exposure [14, 16, 19, 34, 44], of which 52 were newly generated by this study (S2 Table). We restricted our analysis to subjects sampled early (Fiebig stages I-III) and those classified as having a single variant infection. To distinguish the pattern of selection between MSM and HSX founder viruses we used the RELAX test [51] on a multiple sequence alignment of inferred founder strains, without variable loops (which are difficult to align, and could introduce false signal for selection). RELAX is a comparative codon-based phylogenetic framework implemented in HyPhy that formally tests whether selective pressures are intensified or relaxed in one subset of branches (“test”) relative to a “reference” subset of branches, while allowing the strength of selection to vary from site to site in the alignment [51]. Traditionally, the intensity of selection is measured by estimating the ratio (ω) of rates at which nonsynonymous (dN) and synonymous substitutions (dS) are fixed [52], with an excess of dN (ω > 1) an indicator of diversifying positive selection [53]. Here, the point estimates of ω distributions for MSM and HSX branch sets under the Partitioned Exploratory model assigned more codon sites in the HSX lineages to the positively selected category (5.4% [5.0–6.4%] in HSX vs 2.6% [2.3–2.9%] in MSM), although inferred that selection on these sites was stronger in MSM (ω = 15.8 [14.4–17.5] in MSM vs ω = 9.2 [8.2–9.6] in HSX. Therefore HSX founder sequences are subject to broader, albeit weaker, diversifying selective pressure than their MSM counterparts.

**Fig 5:**
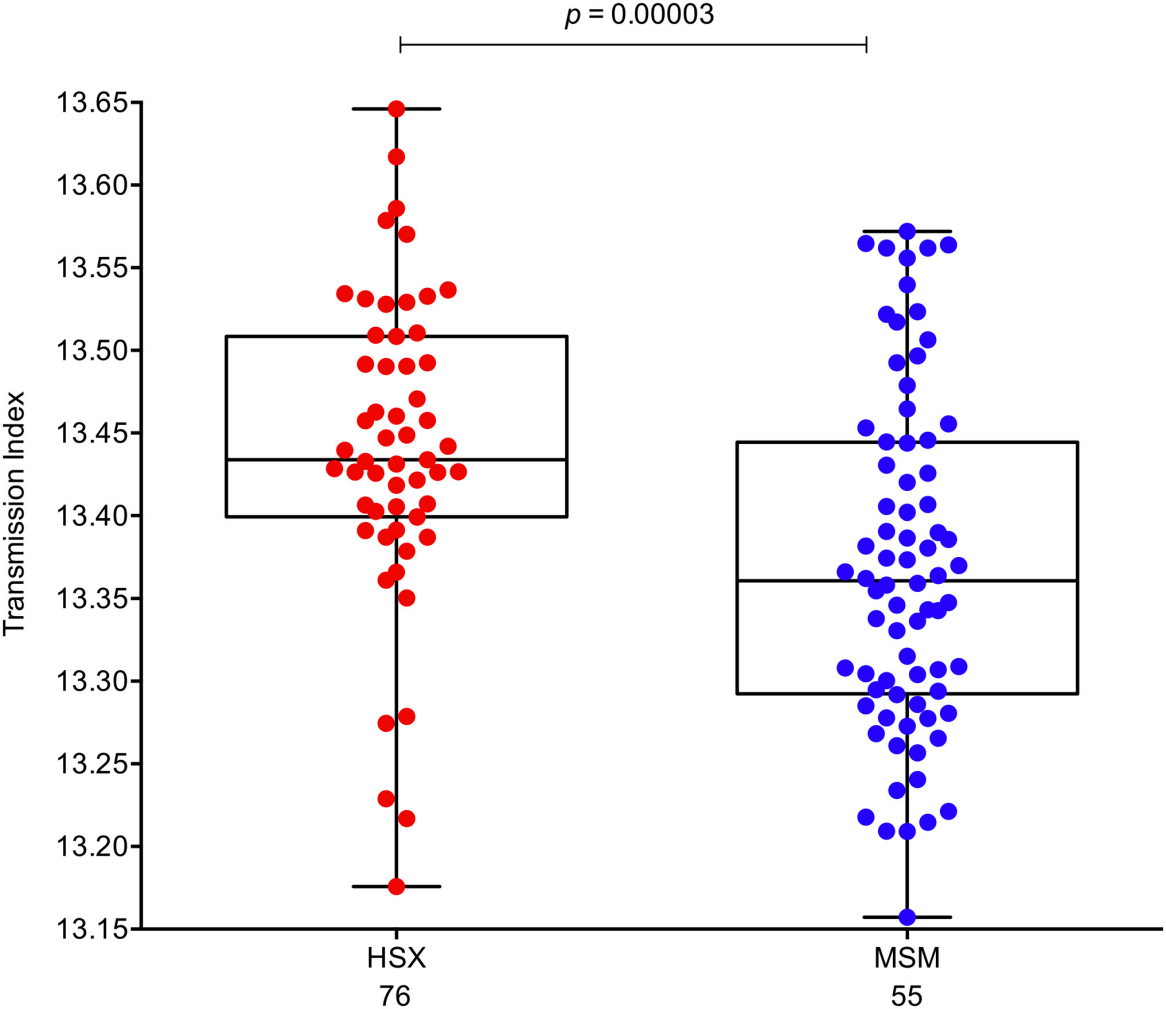
Selection bias intensified for HSX founder viruses compared to MSM founder viruses. The transmission index of a sequence was calculated using logistic regression with model weights taken from [12]. Black lines represent the median transmission index for the two risk groups. The overall transmission index of HSX (red circles) viruses is significantly higher than from MSM (blue circles) founder viruses (*P* = 0.00003, Mann-Whitney two-tailed test). The number of subjects in each category is denoted under each group.

To determine whether other differences exist between HSX and MSM founder viruses we next compared their ‘transmission index’, which was shown to be predictive of which sequence in the donor will establish infection in the recipient [12]. In this recent study examining 137 heterosexual transmission pairs Carlson et al. revealed the preferential selection of viruses exhibiting a more wild-type or consensus-like sequence, perhaps reflective of an optimal HIV-1 genome or one exhibiting higher replicative fitness [12]. We hypothesized that the elevated risk of infection among MSM compared to HSX would result in reduced transmission selection bias, or as such a lower transmission index, upon MSM transmission. Using model weights taken from Carlson et al. [12], we indeed observed significantly lower transmission indices among MSM founder viruses compared to HSX founder viruses (*P* = 3 x 10^−5^, Fig 5). These data indicate that founder viruses from HSX are more closely related to a clade B consensus sequence, are likely to exhibit higher transmission fitness, and are more likely to have undergone proteome-wide selection at the transmission bottleneck as compared to their MSM counterparts, results consistent with the RELAX analysis. Taken together, these data suggest that viral populations from HSX and MSM infections are exposed to distinct selective pressures upon transmission.

### Previously identified founder signature sites are enriched in HSX infection

Recently, Gnanakaran and colleagues reported on a number of sequence motifs in HIV-1 Env associated with founder viruses [34]. In particular, the presence of a histidine at position H12 and the absence of a potential N-linked glycosylation site (PNGS) at position N415 were found to be selected in acute versus chronic viruses. Throughout this study we used the convention of “!” to express the loss of an amino acid at that position. For instance, mutating away from Asn at position 415 would be expressed as! N415. We investigated whether any of these 30 previously identified signature sites were enriched in HSX or MSM founder viruses in our dataset of 131 subtype B sequences. We applied a phylogenetically corrected logistical-regression model [54, 55], and employed a false-discovery rate approach (FDR) to account for multiple comparisons [56]. Adopting a q-value cutoff is critical in this study as thousands of tests were conducted. We generally chose a relatively high q-value cut off in our initial analysis; thus we expect approximately 20% of our sites from our first round of analysis to be by chance.

From this analysis, 3 of the 30 previously identified sites in Env were found significantly enriched in HSX founder viruses: R192 and N362 with a *q* value <0.2 and R633 with a *q* value <0.3 (Table 2; nomenclature denotes cohort consensus residue and HXB2 numbering). More specifically, for residues R192 and N362 we observed selection for maintenance of a consensus residue in HSX founder viruses and at residues N362 and R633 we observed selection away from the non-consensus residue lysine (K) in HSX founder viruses. R192 is located at the base of the V2 loop and Gnanakaran et al. previously reported a loss of arginine (R→!R) in chronic viruses [34]. Similarly, they observed N362 mutating away from an asparagine (N→!N) while at residue R633 the pattern of R→!R was found enriched during chronic infection [34]. This analysis demonstrates that some of the previously found signature patterns of founder viruses may be primarily driven by HSX transmission events.

**Table 2:** Previously described signature sites enriched in HSX Founder viruses.

Predictor Variable^a^	Target Position (HXB2 #)^b^	Target Amino Acid^c^	Target Consensus	Direction ^d^	*p* value	*q* value	Mutation Direction	Domain
HSX	R192	R	R	Adapted	0.0039	0.142	!R—> R	V2 Base
HSX	N362	N	N	Adapted	0.0017	0.1199	!N—> N	C3; PNGS
HSX	N362	K	N	Non-Adapted	0.00106	0.2312	K ->! K	C3; PNGS
HSX	R633	K	R	Non-Adapted	0.0128	0.2312	K ->! K	gp41

Using a phylogenetically corrected logistical-regression model we identified whether any previously published signature sites [34] were associated with HSX mode of transmission. All associations identified were corrected for multiple compairsons with sites with *q*-values <0.3 displayed.

^a^ Predictor Variable: The risk behavior tested. In this case HSX mode of transmission.

^b^ Target Position: The position and identity of the target variable in HXB2 numbering with cohort consensus amino acid listed.

^c^ Target Amino Acid: The position and identity of the target amino acid.

^d^ Direction: Adapted: amino acid is positively correlated with respect to HSX mode of transmission, Non-Adapted: amino acid is present only when risk group is absent

### Identification of novel signature sites in Env associated with mode of transmission

Given the RELAX results, which identified a large number of sites under weak positive selection in the HSX group but a small number of sites under strong positive selection in the MSM group, we next conducted an unbiased search under a phylogenetic corrected framework to identify additional signature sites positively or negatively associated with MSM or HSX transmission. Although all associations identified below using one variable (i.e. HSX) were significant at P<0.05 when using the opposite variable (i.e. MSM), the models are nonetheless distinct and did identify different associations at the q-value cutoffs. From this analysis 7 sites (8 residues) were statistically associated with HSX founder viruses at a *q* of <0.2 (Q389, P724, A823, V832, R845 and A854) or <0.4 (K617, Table 3). All of these sites were found within the gp41 domain with the exception of Q389. Using MSM as the predictor variable revealed 16 sites (17 residues) that were statistically associated with MSM founder viruses at a *q* of <0.2 (T283, K343, Q389, E429, P724, E735, F752, R770, A823, H842, R845) or <0.4 (I165, N362, T465, G471, M518; Table 3). From these MSM-associated sites, 8 are within gp120 while the remaining 8 are within the gp41 domain.

**Table 3:** Signature sites identified between MSM and HSX Founder viruses in Env using a phylogenetic corrected method.

Predictor Variable ^a^	Target Position (HXB2 #) ^b^	Target Amino Acid ^c^	Target Consensus	Direction ^d^	p value	q value	Association Conditions ^e^	Mutation Direction	Domain
MSM	I165	I	I	Non-Adapted	0.00135	0.36973		I ->! I	V2 Loop
MSM	T283	I	T	Non-Adapted	0.00082	0.13190	E275@K	T ->! I	C2 region; CD4 contact
MSM	K343	E	K	Adapted	0.00085	0.13190	N135@E, L515@L	!E -> E	C3 region
MSM	N362	N	N	Non-Adapted	0.00099	0.36973		N ->! N	C3 region
MSM	Q389	P	Q	Non-Adapted	0.00003	0.04125	P417@P	Q ->! P	V4 loop (Beside Lectin DC-SIGN BS)
HSX				Adapted	0.00006	0.04150		!P -> P	
MSM	E429	Q	E	Non-Adapted	0.00026	0.07752	T189@N, K304@R, E347@G	E ->! Q	C4 Region; CD4 contact
MSM	T465	N	T	Adapted	0.00365	0.33233	E293@Q, N404@S, T467@T, H721@H, A792@I	!N -> N	V5 Loop
MSM	G471	A	G	Adapted	0.00048	0.36973		!A -> A	C5 region; CD4
MSM	M518	M	M	Non-Adapted	0.00156	0.36973		M ->! M	gp41; fusion peptide
HSX	K617	K	K	Non-Adapted	0.00215	0.31619	F317@L, M426@R, R444@R	K ->! K	gp41 fusion domain
MSM	P724	P	P	Non-Adapted	0.00013	0.07103	N136@N, T139@T, N463@E	P ->! P	cytoplasmic tail; Kennedy epitope
HSX				Adapted	0.00007	0.04150		!P -> P	
MSM	E735	E	E	Adapted	0.00199	0.19669		!E -> E	cytoplasmic tail; Kennedy epitope
MSM	F752	F	F	Adapted	0.00100	0.13190	S209@S	!F -> F	cytoplasmic tail
MSM	R770	R	R	Adapted	0.00018	0.07103	T139@T, L856@Q	!R -> R	cytoplasmic tail; LLP-2 amphipathic helix
MSM	A823	G	A	Adapted	0.00128	0.13803	N396@E, Q834@Q	!G -> G	cytoplasmic tail
HSX				Non-Adapted	0.00111	0.18648		G ->! G	
MSM	A823	A	A	Non-Adapted	0.00128	0.13803	N396@E, Q834@Q	A ->! A	cytoplasmic tail
HSX				Adapted	0.00111	0.18648		!A -> A	
HSX	V832	I	V	Adapted	0.00082	0.18648	D412@N, T676@S	!I -> I	cytoplasmic tail; LLP-1 amphipathic helix
MSM	H842	N	H	Adapted	0.00040	0.09374	P724@Q, E275@K, N461@D	!N -> N	cytoplasmic tail; LLP-1 amphipathic helix
MSM	R845	T	R	Non-Adapted	0.00098	0.13190	I6@I, E87@A, N141@A, T818@T	R ->! T	cytoplasmic tail; LLP-1 amphipathic helix
HSX				Adapted	0.001017	0.186479	I6@I, N141@A	!T -> R	
HSX	A854	A	A	Adapted	0.0005	0.186479	T413@N, D621@D	!A -> A	cytoplasmic tail; LLP-1 amphipathic helix

^a^ Predictor Variable: The risk behavior MSM or HSX.

^b^ Target Position: The position and identity of the target variable in HXB2 numbering with cohort consensus amino acid listed.

^c^ Target Amino Acid: The position and identity of the target variable.

^d^ Direction: Adapted: amino acid is positively correlated with the risk group, Non-Adapted: amino acid is present only when risk group is absent.

^e^ Association Conditions: The conditions under which the p-value was calculated. Models are built using forward selection, so these predictor variables were added to the model prior to the addition of the site. Sites are labeled with HXB2 numbering and residues correspond to cohort consensus

Four of these sites (Q389, P724, A823 and R845) were found to overlap in both analyses and thus were found to be under significant, but opposing, selection in both HSX and MSM founder viruses. For example, at position Q389, the presence of proline is positively associated with HSX founder viruses while conversely the absence of proline is associated with MSM founder viruses. Specifically, only 1% (1 subject) of MSM sequences contain a proline at position Q389 while 20% (11 subjects) of HSX sequences contain this proline substitution. This Q389 signature site was only seen as significant when taken into account its association with a strongly covarying and highly conserved proline at residue P417 (*q =* 0.086; Table 3). Residue Q389 is located in the α4 helix of the V4 loop in relatively close proximity to the CD4-binding loop [57], and also neighbors the lectin DC-SIGN binding site (N386-T388). Selection here for the bulky hydrophobic residue proline might result in conformational changes in the pocket of the V4 loop and affect virus entry. Notably, this site has previously been found to be under positive selection, with Q389P variants specifically found to reduce HIV-1 replication capacity as well as increase neutralization resistance 15-fold to both b12 and sCD4 [58].

Within the sites identified with MSM as the predictor variable we observed a cluster of sites spatially concentrated (<15 Å) around the CD4 binding loop with at least 6 of the 8 MSM signature sites identified in gp120. Namely I165, T283, N362, Q389, E429, and G471, have previously been shown to alter HIV-1 replication, CD4 dependence and/or lie proximal to the CD4 biding site [57–69]. The identification of signature sites specific to either mode of sexual transmission, further supports that HSX and MSM transmissions are undergoing distinct selection pressures.

### Impact of chronic virus residue frequencies on transmission signature sites

Finally, we sought to determine whether the aforementioned HSX and MSM signature sites described in Table 3 might simply reflect residue frequency differences in the respective HSX and MSM chronic ‘donor’ populations. To assess this we compared our acute sequence data to a panel of chronic viruses comprising over 1300 SGA/S sequences derived from 59 subjects with known HSX or MSM modes of transmission [34] (described as Dataset 4 in Methods). From the newly described signatures we found 12 sites (16 residues) (K343, N362, Q389, E429, T465, K617, E735, A823, V832, H842, R845, A854) that could be influenced by similar residue frequency differences between HSX and MSM chronically infected individuals (S5A Fig). In each of these cases the observed trend in founder viruses is mirrored at chronic infection. For instance, K343E we previously found to be associated with MSM where the presence of glutamic acid (E) is significantly higher in MSM founder viruses compared to HSX founder viruses. However, examination of chronic viruses also revealed the same significant trend where K343E is higher in MSM viruses compared to HSX viruses (*P* = 0.026, Chi-Square test). Thus, the higher frequency of K343E could be driven in part by its higher frequency in chronic circulating strains. Conversely, for 8 of the signature sites (9 residues) (I165, T283, G471, M518, P724, F752, R770 and R845) there was no evidence supporting an influence of differences in chronic residue frequencies impacting the transmission of these variants (S5B Fig). For example, at position P724 in gp41, there is clear selection for a proline (P) in HSX founder viruses while in circulating chronic HSX viruses the frequency of proline was significantly lower than in MSM viruses (*P* < .0001, Chi-Square test). Therefore, the presence of proline appears to be strongly selected for at transmission during HSX infection. Although it is difficult to decipher the origin of these selection events, since higher chronic frequencies could be similarly driven by differences selected at the time of transmission, at least 9 residues are strongly supportive of selection occurring upon transmission followed by regression during chronic infection. Thus, taken together we hypothesize that some of these variants may modestly improve overall fitness and hence be selected for during transmission and conversely selected against (or are neutral) over the course of ensuing chronic infection.

## Discussion

In the present study we build upon a large body of work characterizing acute HIV-1 infection by shedding new light on the genetic properties of founder viruses distinguished by the two primary risk groups responsible for driving the global HIV-1 pandemic. Development of an average pairwise Hamming distance (APHD) approach, benchmarked to SGA/S data as the gold standard method, enabled us to distinguish between single and multiple founder virus infections using deep sequencing data. Application of this approach can also be extended to other genomic regions other than *env* (such as *gag* and *pol*) to assess the complexity of founder virus populations with the caveat that the sensitivity may vary. More importantly, these data demonstrated that the majority (83%) of MSM infections in our cohort exhibited a single founder virus—levels similar to those typically characteristic of HSX infections. Further characterization of these data demonstrated that HSX founder viruses do, however, appear to be under different selective pressures than MSM founder viruses, with HSX founder viruses subject to broader, albeit weaker, diversifying selective pressure than their MSM counterparts. Distinct genetic footprints were also found to be specific to HSX and MSM founder virus populations, supporting discrete selection pressures exhibited by each mode of sexual transmission.

The increasing use of next-generation sequencing has led to the development of specialized computational tools to reconstruct the viral haplotypes that constitute the quasispecies within a single host [70–74], and other methodological approaches have been used to screen for dual infection from deep sequencing data [75]. Although such tools improve our ability to probe the viral diversity they still suffer from a high rate of false positives [76]. As such, our method while based on a sliding window approach exhibits a low-error rate and is easily integrated into our existing data analysis pipeline [77]. While our estimate of the rate of multiple-variant infections in MSM (17%) is lower than the upper range of literature estimates (36–41%) [14, 19] it is not dissimilar from other published reports. Comparative full-length Env analyses have reported frequencies of only 11–14% of multiple founder viruses in MSM [44, 48], with 25% of MSMs in the STEP trial exhibiting multiple founder virus infections [78]. Additional reports using partial regions of Env demonstrated rates of only between 7–9% [79, 80]. One possible source of rate estimate discrepancy could be the inclusion of subjects sampled following peak viremia since immune-induced adaptations, including CD8+ T cell viral escape mutations, APOBEC-induced mutations or recombinants, selected during this window may distort the Poisson distribution model [81]. In our study of 74 subjects we were able to limit the majority of subjects (88%) to the earliest stages of HIV-1 infection (Fiebig I-III) as compared to rates as low as 60% of subjects in previous studies [14, 19]. Consistent with this hypothesis we found the odds of observing a multivariant infection in MSM was almost four times higher at later Fiebig stages IV-VI than in subjects sampled during earlier Fiebig I-III stages of infection (odds ratio, 3.86; 95% CI, 1.64 to 9.08; Fisher’s exact test, *P* = 0.003). Importantly, although differences in the number of founder viruses could be attributable to compartmentalization in the source, examination of viruses in a cohort of Zambian transmission pairs found no evidence for preferential selection in the donor genital tract [41]. Thus, our findings support that although the risk of HIV-1 acquisition is significantly greater in MSM, this increased risk is not reflected by the transmission of an increased number of founder viruses.

While it is clear that various bottlenecks can limit the number of HIV-1 founder viruses successfully transmitted from a diverse, chronically infected donor (as recently reviewed in [82]), factors determining what viruses survive the bottleneck are not well understood. A recent study by Carlson et al. examining 137 heterosexual transmission pairs revealed the preferential selection of viruses exhibiting a more wild-type or consensus-like sequence, perhaps reflective of an optimal HIV-1 genome or one exhibiting higher replicative fitness [12]. Termed here a ‘transmission index’, this effect was more pronounced in female-to-male transmission compared to male-to-female transmission, and the effect could be attenuated by donor viral load and presentation of genital ulcers or inflammation (GUI). In our current study we also observed that HSX founder viruses exhibit significantly higher transmission indices than MSM founder viruses. In the absence of any donor sequence information, these data support a model in which there exists stronger selection forces, or increased opportunity for selection, upon the incoming viral quasispecies during HSX versus MSM transmission to optimize for wild-type or high-fitness variants for successful dissemination. Thus, this data fits with the prediction of modeling transmission as a binomial mixture process in which infection risk is inversely correlated with the strength of selection [12]. Given the elevated risk of infection in MSM compared to HSX we expected that the selection bias experienced by MSM founder viruses to be less stringent than that observed for HSX transmission. Such an overall reduced selective bias may make infection more conducive to even subtly weaker viruses. However, these viruses may need to optimize for enhanced CD4 binding in order to gain an advantage and successfully disseminate. Hence, the MSM-selected cluster of sites around the CD4 binding site may be evidence for such a scenario. Moreover, the differences in the selection bias at the transmission bottleneck may transcend to differences in clinical outcomes with reduced bias resulting in increased virulence with faster rates of reversion leading to higher fitness viruses emerging. On the other hand increasing the transmission selection bias may incite founder viruses that are optimized for increased fitness resulting in higher viral loads and poorer clinical outcomes in the newly infected individual.

Given that the majority of our HSX subjects were men (79% of our 131 founder virus dataset), and that within MSM the risk from unprotected receptive anal intercourse is >10 fold higher than for insertive anal intercourse [20], our study is effectively comparing penile (HSX) versus rectal (MSM) receptive routes of HIV-1 transmission. As such, our data would suggest that the rectal route may exert less selection pressure upon the incoming viral quasispecies than transmission through penile exposure, consistent with model predictions [12]. In the rhesus macaque model, even after penile exposures to a high dose SIV inoculum only a single variant founder population establishes infection [83, 84], while high dose intra rectal exposures are associated with greater numbers of founder viruses [85]. The rectal compartment is highly vulnerable to HIV-1 transmission with a single more fragile layer of columnar epithelium separating the lumen from the lamina propria as compared to the stratified squamous epithelium found in the ectocervix and vagina or the inner foreskin and the glans epithelia of the penis in uncircumcised men [86]. This, coupled with the density of HIV-1 target cells populating the rectum such as activated CD4+ T cells, macrophages and dendritic cells, may contribute to the greater risk of HIV-1 transmission associated with men who have receptive anal sex with men compared with HSX transmission in men or women [87, 88], but may also result in relaxation of the selective pressures upon the incoming quasispecies. Indeed, a briefer and narrower eclipse phase has been observed for HIV-1 infections acquired rectally compared to those acquired through the vaginal or penile tissues [83, 89, 90] where local viral expansion is necessary before the dissemination of infection to the bloodstream. Thus, MSM viruses may not need to undergo the same level of selection that HSX viruses must endure for successful replication and systemic dissemination.

Given previous studies have espoused differences in variable loop length and potential N-linked glycosylation site count in the transmitted virus for HIV-1 subtypes A and C [21, 24, 32], although less clear in subtype B infections [31–33], we searched for any association between variable loop diversity and mode of transmission. Detailed analyses of the variable loops revealed only one such putative association with MSM founder viruses encoding a more compact V2 loop compared to HSX founder viruses (mean of 40.9 residues for MSM and 42.5 for HSX) although such an effect did not reach statistical significance after correcting for multiple comparisons. While one study has identified through the comparison of acute versus chronic HIV-1 sequences signature mutations associated with founder viruses [34], our study extends these findings by identifying an array of genetic signatures that may be distinct between MSM and HSX risk groups. Interestingly, the majority of residues found to be associated with HSX risk were located within the gp41 domain, and in particular within the cytoplasmic tail. At residue K617 we observed maintenance of a consensus lysine residue where mutations at this position of the gp41 fusion domain have been shown to significantly reduce viral entry [91]. This unusually long and highly conserved domain of approximately 150 amino acids modulates a diverse array of functions, including viral replication, Env incorporation into virions, and intracellular trafficking and endocytosis to regulate levels of Env surface expression (reviewed in [92]). The C-terminal half of the cytoplasmic domain is characterized by the presence of three structurally conserved α-helices designated lentivirus lytic peptide 1 (LLP-1), LLP-2, and LLP-3 [93–95]. Notably, three of the HSX signature residues (V832, R845 and A854), where we saw selection for the consensus residue in HSX, are located within the LLP-1 region, which is associated with Env incorporation into virions [96, 97]. Thus, these specific signature sites within gp41 may influence Env virion incorporation levels and viral entry, thus increasing transmissibility. It is also conceivable that these specific mutations may alter the conformation of the envelope trimer in such a manner that is favored for initial infection. Notably, many of the strongest transmission signature sites observed by Gnanakaran et al were also in the cytoplasmic domain in addition to enrichment for histidine at residue H12 in the signal peptide [34], the later of which has been demonstrated to increase Env incorporation and infectious titers [35]. Regardless of the precise mechanism, these data support a role for selection upon the cytoplasmic domain of HIV-1 gp41 during transmission.

**Fig 6:**
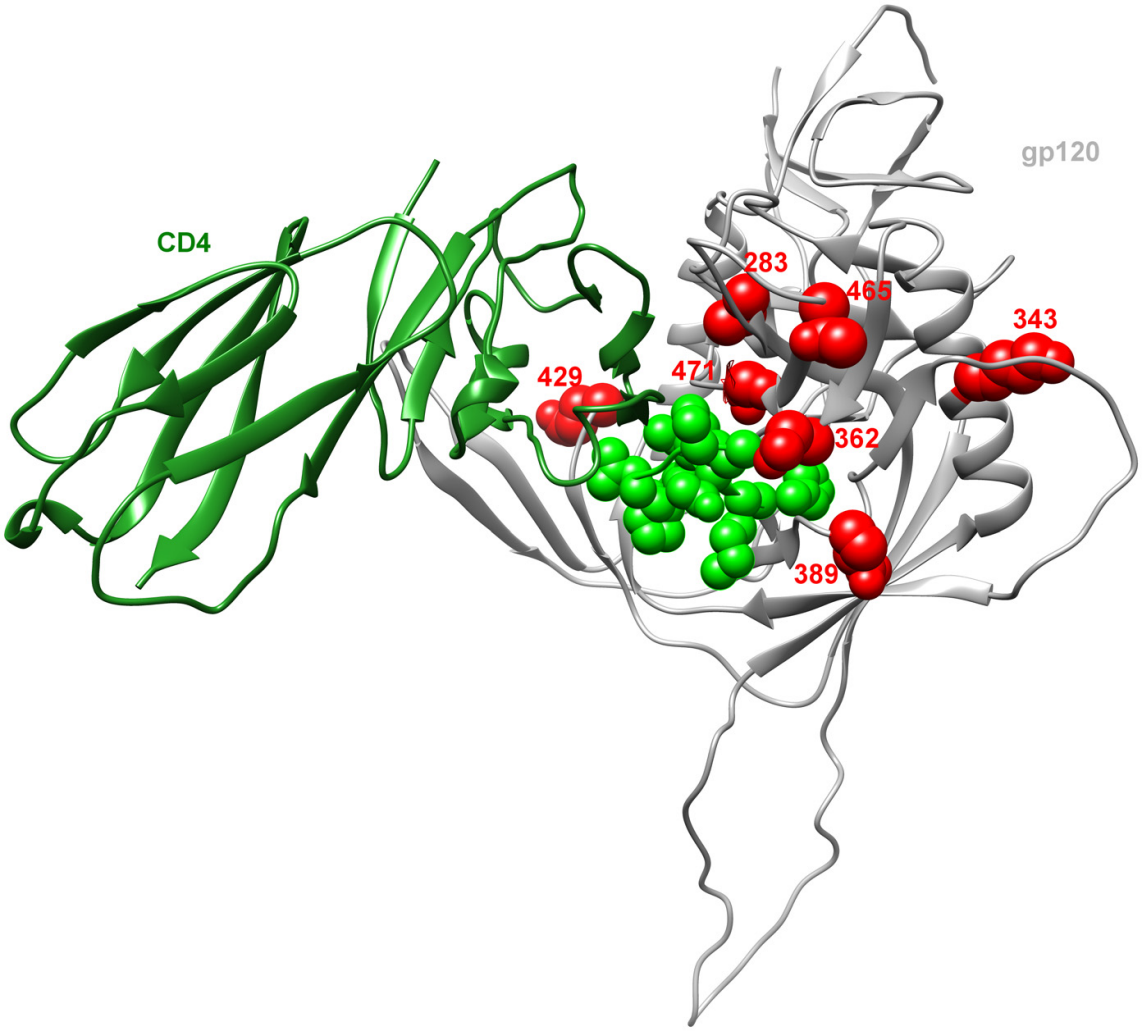
Mapping of signature sites on the three-dimensional structure of gp120 shows clustering around the CD4-binding site. A ribbon representation of the crystal structure from the JRFL gp120 molecule (grey) bound to CD4 molecule (green) (PDBID: 2B4C). The CD4 binding site is highlighted in transparent green while signature sites 283, 343, 362, 389, 429, 465 and 471 are all depicted as *red* space-filling residues.

In contrast, nearly half of the residues associated with MSM risk were located in gp120 with six residues (T283, N362, Q389, E429, T465, G471) clustered around the CD4-binding pocket with the potential to influence CD4 binding (Fig 6). In addition to residue Q389 described earlier, which is located in close proximity to the CD4-binding loop [57], position T283 has been shown to affect CD4 binding site exposure and CD4 binding of gp120s derived from brain and other tissues [60]. Similarly, presence of the N362 PNLG site in the C3 region has been shown to enhance CD4 binding to gp120 as well as cell-cell fusion [68, 98], potentially reducing CD4 dependence by stabilizing the CD4-bound confirmation of gp120 [68]. Meanwhile, at residue E429 located in the C4 domain of gp120 we observed selection for glutamine (E429Q) where prior work has identified this residue as being critically important for the binding of CD4-blocking MAbs [65] and implicated in altering resistance to the entry inhibitors BMS-806 and #155 [63], as well as enhancing HIV-1 replication *in vitro* [59]. Within the V5 loop, residue T465 has also been associated with a neutralization-resistant phenotype [99], while finally at residue G471 where we observed selection for an alanine (G471A) the variants G471R/E have been shown to impart resistance towards CD4 mimetic compounds [61]. Thus, many of the signature sites identified in MSM in gp120 may influence gp120-CD4 interactions for enhanced interactions with CD4.

A limitation of this study is the inclusion of subjects designated as source plasma donors (SPD). These subjects had limited behavioral information available but as part of routine blood-banking practice underwent extensive questioning for HIV-1 risk behaviors and denied having sex for money, homosexual activity or i.v. drug use. Nevertheless, self-reporting of risk behaviors among paid plasma donors is imperfect and it is plausible that some subjects whom were designated as belonging to the HSX risk group as previously categorized [19, 34] may have additional risk behaviors. However, the exclusion of all SPD subjects from our comparative analysis indicates that the frequency of multi-variant transmission between HSX and MSM transmission remained the same (22% vs. 25%, odds ratio, 1.16; 95% CI, 0.66 to 2.06; Fisher’s exact test; *P* = 0.66). The reported higher transmission indices for HSX founder viruses also continued to be significant when compared with MSM founder viruses (*P =* 0.0007, Fisher’s exact test). Thus, the overall study findings are unlikely to have been influenced by the inclusion of this subject group alone.

While HIV-1 acquisition in MSM may be immunologically and virologically distinct from that of heterosexual exposure, we observe no differences in the number of HIV-1 founder viruses. Although the severe HIV-1 transmission bottleneck has a stochastic component in which any reasonably fit CCR5-tropic virus may be capable of establishing productive infection, our data do argue that any selection bias may be comparatively relaxed with ano-rectal MSM transmission, potentially due to the greater frequency of target cells at the site of transmission and the distinct kinetics of virus dissemination [87–90]. Conversely, upon heterosexual exposure we observe an increased bias for consensus-like viruses with a potentially higher replicative fitness that must undergo local viral replication prior to systemic dissemination. In the era of new therapeutic approaches such as AAV delivered HIV-1 inhibitors [100] and effective pre-exposure prophylaxis [101], it remains to be seen whether breakthrough infections will lead to higher fitness viruses being selected for resulting in more severe clinical outcomes, akin to what was observed during the CAPRISA 004 vaginal microbicide gel trial [102]. More accurate estimation of the frequency of multi-variant infection will also aid in evaluating the clinical impact that infection with multiple variants has on disease progression as a number of studies have reported associations with increased viral load [103–105] and faster CD4+ T-cell decline [106, 107] resulting in a shorter time to AIDS. Finally, given the recent application of discerning the number of founder viruses as a measurement of relative protection from infection [108, 109], more critical delineation of the genetic make-up and complexity of the founder virus population may be important towards the development of an effective HIV-1 vaccine.

## Materials and Methods

### Study subjects

Plasma samples were obtained from subjects with acute or early HIV-1 infection enrolled in HIV-1 cohorts in Berlin, Germany, and Massachusetts, California, North Carolina, and South Carolina, USA. The clinical and sociodemographic characteristics of the study participants can be found in S1 Table and Fiebig staging criteria are described in Supplementary Materials and Methods in S1 Text. All study subjects gave written informed consent and plasma collections were performed with local institutional review board and other regulatory approvals. This study was approved by the Institutional Review Board of Massachusetts General Hospital.

### PCR amplification and 454 sequencing

HIV-1 was PCR amplified and 454 sequenced using a nested RT-PCR with 3 amplicons overlapping the genome. Briefly, viral RNA was isolated from 1ml of plasma using the QiAmp Viral RNA Mini Kit (Qiagen, Valencia, CA) and RT-PCR of near full-length HIV-1 genomes performed using nested-PCR primers specific for gag, pol and 3′ half of the viral genome (see S1 Text and S3 Table). Pooled PCR products were prepared for sequencing on the 454 Genome Sequencer Junior (Roche) using the Nextera DNA Sample Prep Kit and data processed performed using our previously published sequence analysis pipeline [77, 110] (see S1 Text).

### Average pairwise Hamming distance measurement of 454 sequence diversity

Following alignment of cleaned reads to the consensus assembly sequence a sliding window approach was used to collect all reads that covered a window of 120bp with a step size of 21bp. We then calculated the pairwise Hamming distance (HD) (defined as the number of base positions at which the genomes differ, excluding gaps) for all reads and averaged this value over all windows to obtain the average pairwise Hamming distance (APHD). To formalize the criteria and evaluate the APHD approach for its discriminative ability we obtained SGA/S sequence data from studies where each sample had been previously designated as infection by a single virus or infection by multiple viruses [14, 19]. To introduce sequencing errors, a synthetic read dataset was simulated from the SGA/S data using the 454 sequencing error profile from ART a next-generation read simulator [111]. Reads were simulated with varying degrees of coverage in order to achieve the same representative coverage obtained from our deep sequencing data with multiple replicates performed to rigorously assess the uncertainty due to 454-like sampling.

### Discrimination of 454 sequence data for single and multiple founder viruses

To differentiate between single and multiple founder viruses using 454 sequencing reads intra-patient HIV-1 genetic diversity was first characterized as previously described, with nucleotide-phasing information used to distinguish true variants from sequencing errors (see S1 Text and S6 Fig). The mean APHD is calculated using the sliding window approach as previously discussed. Reads were then carefully inspected to rule out factors that might compromise the amount of diversity such as the emergence of CTL escape mutations or early reversions whereby diversity would be restricted to narrow windows specific to known CD8+ T cell epitopes specific to the subject’s HLA and would appear as distinct peaks on the APHD landscape. More specifically, for each individual the optimal “A-list” of CTL epitopes restricted by a subject’s HLA alleles was generated and local haplotype windows were reconstructed across each of the epitopes to assess for any evidence of putative CTL escape mutations. Any windows in which CTL escape mutations were found were further examined and the contribution of this window to the overall APHD score was evaluated to unsure that it did not unduly influence any subject designation as infected by multiple viruses.

### Single genome amplification and sequencing of 3′ half genome

cDNA was serially diluted and amplified as previously described [14, 17, 112], and the amplified products sequenced using a 454 GS Junior (see S1 Text).

### Datasets

A number of previous published datasets were used throughout this study and are numbered accordingly. Briefly, Dataset 1 comprising the SGA/S data collected from 127 acute individuals sampled at varying times post-infection and clinically staged as described by Fiebig et al [113] were used [14, 19] to test the performance of the APHD approach. To explore the relationship between multiplicity of infection and mode of transmission, Dataset 2 encompassing 354 subjects, for whom full-length SGA/S envelope sequences had been generated, encompassing MSM, HSX and IDU transmissions (including the 74 subjects newly deep sequenced during this study) [7, 14, 15, 19, 34, 44, 47, 49, 50] were obtained. From the total of 354 subjects a subset of 131 HIV-1 founder viruses from subjects reporting a sexual exposure were selected [14, 16, 19, 34, 44] (Dataset 3). The criteria for subject selection was restricted to clade B infections sampled early (Fiebig stages I-III) and included only subjects previously classified as being infected with a single virus (subjects listed in S2 Table). Dataset 4 included chronic samples obtained from Gnanakaran et al [34]. This dataset contained over 1300 SGA/S sequences derived from 59 subjects with known exposure status defined as HSX or MSM. These sequences were from individuals who were not on anti-retroviral therapy, and infected for a minimum of two years and represented clade B infections predominantly collected in the United States.

### Mathematical model of random evolution

PoissonFitter was used to test the hypothesis that a single virus establishes infection [81]. PoissonFitter performs two tests: one test is based on the fit of the Poisson model to the frequency distribution of the Hamming distance observed in each sample; the other is a topological test to verify that observed frequencies are distributed according to a star-like phylogeny (for this test, no formal statistic is available and consequently no *p*-value is obtained). In this model the main assumption is that a single founder virus evolves under neutral evolution, generating a star-like phylogeny, with a distribution of mutations conforming to a Poisson distribution [13, 14].

### Identification of signatures sites in Env using a phylogenetically corrected approach

We used the phylogenetically corrected logistic regression to identify sites positively or negatively associated with MSM or HSX state [54, 55]. Briefly, this approach uses standard logistic regression, with the modification that information from the phylogeny is used to inform the bias parameters. Rather than assuming the sequences are independent and from the same distribution, the phylogenetically corrected logistic regression model assumes the sequences are drawn from a known phylogenetic structure. Using this structure separate phylogenetically corrected logistic regression models were learned from each amino acid at each site. Phyml v3.0 [114] was used to infer the phylogenetic structure, using all sequences available in this study. Using this structure, separate phylogenetically corrected logistic regression models were learned for each amino acid at each site. For “indirect” models, only a single feature representing MSM or HSX status was used. For “direct” models, forward selection was used to learn a model that possibly included covariation from other sites in addition to MSM or HSX status. Note that although MSM = 1-HSX, the models are distinct and may identify different associations. In our case, while the association’s differed when using *q*-value cutoff, all associations identified using one variable were significant at P<0.05 when using the opposite variable. The *q*-value is the minimal false discovery rate that adjusts for multiple tests [56]. The appropriate choice of *q*-value threshold is context specific and depends on how the results will be interpreted. In the present study, we typically report all tests where *q* is <0.2 (implying that we expect 20% of reported tests to be false positives) but sometimes report higher *q* values to include sites in a hypothesis-raising framework. The associations identified in this study are referred to as *adapted* and *nonadapted* forms. Adapted forms (commonly referred in many studies as escape variants) are amino acids significantly enriched for in the presence of the risk behavior in question. Nonadapted forms (also commonly called wild-type or susceptible forms) are amino acids significantly depleted in the presence of the risk behavior.

### Detection of selection under a phylogenetic framework

To distinguish the pattern of selection between MSM and HSX founder viruses we used a comparative codon-based phylogenetic framework test implemented in HyPhy that formally tests whether selective pressures are intensified or relaxed relative to a subset of branches [51]. In this case we assessed whether selective strength on the test subset of branches is compressed toward or repelled away from neutrality, relative to the reference subset of branches. Under this analysis we labeled HSX viruses as reference branches while MSM viruses were labeled as test branches. Internal branches were labeled as MSM or HSX if all of their descendants were also labeled MSM or HSX, respectively. The Null model, which forces HSX and MSM to share the same selective regime can be rejected in favor of the Partitioned Exploratory model (p = 0.009, Likelihood Ratio Test). The Partitioned Exploratory model is merely the model where the “test” and “reference” branches in the tree are endowed with completely independent discrete distributions of omega parameters. The Alternative model, which is a restriction of the Partitioned Exploratory model forces the proportions of sites under different types of selection to be the same can be similarly rejected (p = 0.008). All confidence intervals listed are 95% profile likelihood approximations.

### Transmission index

In the context of heterosexual linked transmission pairs, we previously trained a model that estimates the probability that any particular amino acid will be transmitted from a donor to a recipient [12]. Although this model was trained grouping together all residues at all sites using a generalized linear mixed model, we showed that a simple extension to full sequences, which we called the “Transmission Index” was predictive of which sequence would establish infection. Here we computed the transmission index of each founder virus in MSM vs HSX founder viruses using logistic regression, with model weights taken from Table 2 of [12]. Amino acid conservation and covariation was taken from the clade B envelope sequence data of [115].

### Statistical analysis

All statistical analysis was performed using JMP Pro, version 12 (SAS Institute). Descriptive measures were used to summarize the data. Continuous variables were summarized using median and inter quartile range (IQR); categorical variables were summarized using frequency and percent (%). Chi-square and Mann-Whitney tests were used to compare categorical and continuous variables between the study groups, respectively.

## Supporting Information

S1 Text Supplementary materials & methods. (DOC)

S1 Fig High *env* diversity in subject 882283 reflecting a heterogeneous infection suggestive of infection by at least 4 founder viruses. **(A)** Heatmap from the 454 sequencing data illustrating a diverse number of sites throughout the 3′ half of the genome showing up to 40% codon diversity. **(B)** The APHD plots showing a mean APHD of 0.306 (red line) and standard deviation (dotted black line) demonstrating a high level of diversity across the 3′ half of the HIV-1 genome. **(C)** SGA sequences displaying a phylogeny (left) revealing infection by at least four viruses with inter-lineage recombinants. Founder virus lineages are color-coded while recombinant sequences are shown by green symbols. Highlighter plots (right) compare sequences for each subject’s sequence set to an intrasubject consensus (uppermost sequence) and depict the pattern of nucleotide base mutations. Subject 882283 was viral RNA positive but Western blot negative (Fiebig stage II/III of infection).
(TIF)

S2 Fig High *env* diversity in subject 702865 reflecting a heterogeneous infection suggestive of infection by at least 4 founder viruses with inter-lineage recombination. **(A)** Heatmap from the 454 sequencing data illustrating a diverse number of sites throughout the 3′ half of the genome showing up to 40% codon diversity. **(B)** The APHD plots showing a mean APHD of 0.593 (red line) and standard deviation (dotted black line) demonstrating a high level of diversity across the 3′ half of the HIV-1 genome. **(C)** SGA sequences displaying a phylogeny (left) revealing infection by at least four viruses with inter-lineage recombinants. Founder virus lineages are color-coded while recombinant sequences are shown by green symbols. Highlighter plots (right) compare sequences for each subject’s sequence set to an intrasubject consensus (uppermost sequence) and depict the pattern of nucleotide base mutations. Subject 702865 was viral RNA positive but Western blot indeterminate (Fiebig stage IV of infection).
(TIF)

S3 Fig High *env* diversity in subject 574194 reflecting a heterogeneous infection suggestive of infection by at least 3 founder viruses. **(A)** Heatmap from the 454 sequencing data illustrating a diverse number of sites throughout the 3′ half of the genome showing up to 40% codon diversity. **(B)** The APHD plots showing a mean APHD of 1.718 (red line) and standard deviation (dotted black line) demonstrating a high level of diversity across the 3′ half of the HIV-1 genome. **(C)** SGA sequences displaying a phylogeny (left) revealing infection by at least four viruses with inter-lineage recombinants. Founder virus lineages are color-coded while recombinant sequences are shown by green symbols. Highlighter plots (right) compare sequences for each subject’s sequence set to an intrasubject consensus (uppermost sequence) and depict the pattern of nucleotide base mutations. Subject 574194 was viral RNA positive but Western blot positive with 3 bands (Fiebig stage V of infection).
(TIF)

S4 Fig Comparison of SGA sequences for subject 1051 who displays evidence of infection with multiple founder viruses. SGA sequences derived from this study (red) were compared to sequences derived from Keele et al. [14] in which 3 additional timepoints were sequenced. Previous analyses by Keele revealed infection by at least 4 founder viruses while in this study we found infection by at least 3 viruses [14].
(TIF)

S5 Fig Frequency comparison of signature sites between acute and chronic viruses during HSX and MSM infection. Frequency comparison of previously found signatures sites in our panel of HSX and MSM founder viruses compared to a dataset of chronic HSX and MSM viruses. Chronic viruses comprised 462 SGA/S sequences derived from 24 chronically infected subjects who reported heterosexual as their risk factor for HIV-1 infection and 867 SGA/S sequences from 35 chronically infected MSM subjects. Sites and amino acids examined are listed on the x-axis with the frequency of that amino acid at that position shown on the y-axis. Acute HSX (red bars), acute MSM (blue bars), chronic HSX (red striped bars) and chronic MSM (blue striped bars). **(A)** Sites that show the same frequency trend in chronic and acute infection are depicted. **(B)** Sites that show the opposite trend in chronic and acute infection are shown. Sites that showed a statistically significant difference between the chronic stage of infection at a *P* value of less than 0.05 (Chi-Square test) are indicated by a single *asterisk* (*).
(TIF)

S6 Fig Distribution of point mutation errors found within a plasmid control. **(A)** Distribution of point mutation errors across the HIV-1 genome from 3 independent sequencing runs of an HIV-1 NL4-3 plasmid control. **(B)** Effect of PCR amplification on point mutation variant mismatches across three independent PCR and sequencing runs.
(TIF)

S1 Table Clinical description of cohort study subjects with acute or early HIV-1 infection. (XLSX)

S2 Table A description of the 131 founder viruses used in this study. (XLSX)

S3 Table A list of all primers used to amplify the HIV-1 genome. (XLSX)
