# Supplementary material for: Differences in the Selection Bottleneck between Modes of Sexual Transmission Influence the Genetic Composition of the HIV-1 Founder Virus
Source: PLoS Pathog. 2016 May 10;12(5):e1005619. doi: 10.1371/journal.ppat.1005619 (PMC4862634; doi:10.1371/journal.ppat.1005619)
Supplement: S1 Text — (DOC) [file ppat.1005619.s001.doc]

**S1 TEXT**

**Supplementary Materials & Methods**

**Clinical Staging of Acute HIV-1 Infection**

Timing of infection was estimated based on serological test results as described by Fiebig et al [1]. Briefly, PCR, p24 ELISA and Western Blot test results were used to designate the earliest diagnostic plasma as having been taking during Fiebig I, Fiebig II/III (the absence of 2^nd^-generation EIA test results precluded discrimination between stage II and III) and Fiebig IV (ELISA positive, Western Blot indeterminate). A conservative estimate of days post infection (dpi) by using the maximum value of the 95% confidence interval of the mean duration of each Fiebig stage: Fiebig I = 15 dpi; Fiebig II/III = 25 dpi; Fiebig IV = 31 dpi; Fiebig V = 70 dpi and Fiebig VI >100 dpi.

**Viral RNA Isolation and Quantification**

One ml of plasma was thawed on ice and centrifuged at 14,000 x g for 1.75 hours at four degrees Celsius. The pellet was resuspended in 140 µl of supernatant and the viral RNA was isolated following the protocol for QiAmp Viral RNA Mini Kit (Qiagen, Valencia, CA).. The RNA was eluted in 60 µl of RNA storage solution (Applied Biosystems/Ambion, Austin TX), aliquoted, and stored at -80 degrees Celsius. Quantitative RT-PCR (qRT-PCR) using HIV-1 *gag* SK145 (AGTGGGGGGACATCAAGCAGCCATGCAAAT) and SK431 (TGCTATGTCACTTCCCTTGGTCTCT) primers at 300 nM concentration and the QuantiTect SYBR Green RT-PCR Kit (Qiagen) following the kit’s protocol. The quantification standards consisted of linear, near-full-length, HIV-1 clade B plasmid DNA derived from the pHXB2-RU3 plasmid.

**RT-PCR Nested Amplification**

For each sample, a max of 2,000 copies was used for the initial RT-PCR, depending on which was the smaller volume. Three primer pairs were synthesized to amplify three overlapping regions of the HIV-1 genome (*gag*, *pol*, and 3′ half). All primers are presented in **S3 Table**. Each RT-PCR reaction included 2X reverse transcription (RT) buffer (a buffer containing 0.4 mM of each dNTP, 2.4 mM MgSO_4_), 0.4 µM each primer, and 200 units/µl SSIII Platinum Taq Polymerase High Fidelity (Invitrogen, Carlsbad, CA). The conditions used for the initial RT-PCR differed for the three primer sets. *gag* amplicons were generated with the following PCR parameters: an RT step of 55**°**C for 30 minutes; an initial denaturation step of 94**°**C for 2 minutes; 30 cycles of a denaturation step of 94**°**C for 1 minute, an annealing step of 65**°**C for 30 seconds, and an extension step of 68**°**C for 2 minutes; and a final extension step of 68**°**C for 10 minutes. *pol* amplicons were generated with the following PCR parameters: an RT step of 55**°**C for 30 minutes; an initial denaturation step of 94**°**C for 2 minutes; 30 cycles of a denaturation step of 94**°**C for 1 minute, an annealing step of 56**°**C for 30 seconds, and an extension step of 68**°**C for 2.5 minutes; and a final extension step of 68**°**C for 10 minutes. The 3′ half amplicons were generated with the following PCR parameters: an RT step of 55**°**C for 30 minutes; an initial denaturation step of 94**°**C for 2 minutes; 40 cycles of a denaturation step of 94**°**C for 15 seconds, an annealing step of 60**°**C for 30 seconds; and an extension step of 68**°**C for 5 minutes, and a final extension step of 68**°**C for 10 minutes.

Each second round PCR reaction included 2µl of round 1 product from the respective RT-PCR, 10X Takara Buffer (TaKaRa), 0.6 µM of each primer, 0.25 mM of each deoxynucleoside triphosphate, and 1.25 units/µl of TaKaRa Ex Taq HS DNA polymerase (TaKaRa). *gag* amplicons were generated with the following PCR parameters: an initial denaturation step of 94**°**C for 2 minutes; 30 cycles of a denaturation step of 94**°**C for 1 minute, an annealing step of 65**°**C for 30 seconds, and an extension step of 68**°**C for 2 minutes; and a final extension step of 68**°**C for 10 minutes. *pol* amplicons were generated with the following PCR parameters: an initial denaturation step of 94**°**C for 2 minutes; 10 cycles of a denaturation step of 94**°**C for 1 minute, an annealing step of 58**°**C for 30 seconds, and an extension step of 68**°**C for 2.5 minutes; 20 cycles of a denaturation step of 94**°**C for 1 minute, an annealing step of 58**°**C for 30 seconds, and an extension step of 68**°**C for 2.5 minutes with additional 5 seconds added to the extension per cycle; and a final extension step of 68**°**C for 10 minutes. Finally, 3′ half amplicons were generated with the following PCR parameters: an initial denaturation step of 94**°**C for 2 minutes; 40 cycles of a denaturation step of 94**°**C for 30 seconds, an annealing step of 60**°**C for 30 seconds, and an extension step of 72**°**C for 5 minutes; and a final extension step of 68**°**C for 10 minutes.

For quality control purposes, the products were run on a 1% agarose gel. Positive PCR products were purified using the QIAquick PCR Purification Kit (Qiagen) or the PureLink Quick Gel Extraction Kit (Invitrogen) and concentrations were determined using the Thermo Scientific Nanodrop 8000 spectrophotometer (Thermo Scientific, Nanodrop Products, Wilmington, DE). Amplifications from the same subject were pooled with concentration ratios of *gag*: *pol*: 3′ half being 1:1:3.

**454 Library Construction and Sequencing of Full-Length Genomes**

Samples were fragmented using the transposon-mediated Nextera DNA Sample Prep Kit for 454 per manufacturer’s protocol (Illumina, San Diego, CA). This protocol generates sequences between 300 and 500 basepairs in length. Samples were then purified with a DNA Clean and Concentrator Kit (Zymo Research, Irvine, CA) and adapters and barcodes were added by limited cycle PCR (Nextera). Small fragments were removed using following Roche’s recommended size selection protocol and were quantified using a Promega Quantiflor-ST flourometer (Promega, Madison, WI). After quantification, barcoded samples were pooled at a final concentration of 10^7^ molecules/μl to create the library for sequencing on the 454 GS Junior instrument (Roche, Indianapolis, IN). Emulsion PCR, breaking, and DNA sequencing were performed according to manufacturer’s protocols for Lib-L (Roche). In total a dataset comprising over a million reads comprising 4.1 x 10^8^ bases was attainted generating an average sequence coverage per subject (sequencing reads per site) of 584 ± 240. Whole genome was produced for all subjects with the exception of two in which the *pol* amplicon could not be generated (subjects 494131 and 848017) while for 1059_09 only the 3′ half amplicon could be generated. In each case the number of input template molecules (>2,000 RNA copies) was greater than the fold depth of sequence data achieved. The congruence of variants found between 454 and SGA/S (data not shown) further supports that template resampling did not have an influence on our findings.

**454 Sequencing Data Cleaning Strategy**

First, the data was cleaned by a set of scripts that discarded reads less than 150 base pairs in length and excluded reads containing any ambiguous bases (Ns). Remaining reads were then assembled into a de-novo consensus assembly sequence by AssembleViral454 (AV454) or VICUNA [2, 3]. V-FAT (Broad Institute, <http://www.broadinstitute.org/scientific-community/science/projects/viral-genomics/v-fat>) was used for automated computational finishing and annotation of *de novo* viral assemblies. Alignments to the consensus assembly were generated using Mosaik (version 2.1.73) and reads were corrected for systematic 454 errors such as homopolymer frameshift errors and carry forward and incomplete extension (CAFIE) using Read Clean 454 (RC454). After cleaning, reads were passed to V-Phaser for accurate variant calling. Briefly, V-Phaser uses phase and quality filtering with a probability model that recalibrates quality scores for individual bases to iteratively refine probabilities and to define the threshold required to statistically define a true variant from a sequencing artifact [4]. Read alignments were visualized with IGV (Integrative Genomics Viewer) and subjected to manual inspection to identify and discard any sequencing related artifacts. All consensus genome assemblies and annotations generated as part of this study were submitted to NCBI’s GenBank database under the following accession numbers: KT124741 – KT124814 and the raw 454 deep sequencing data was deposited in the NCBI Sequence Read Archive under study accession numbers SRA270093 and SRA272235 (NCBI BioProject. PRJNA284362).

**Estimation of 454 Deep Sequencing error rate, and sensitivity**

To measure the accuracy of our 454 deep-sequencing protocol, a plasmid (50ng) derived from a stock of HIV-1 NL4-3 containing a mutation in the Gag SL9 epitope was fragmented, barcoded and sequenced. The entire procedure from sample preparation to 454 deep sequencing was repeated three times. In addition to evaluating the source of error from the entire 454 deep sequencing processes the source of error originating from PCR was also examined. For this, the HIV-1 NL4-3 infectious clone was amplified and sequenced. First-round reactions used 2,000 copies of RNA isolated from a stock NL4-3 virus. Two μl of the dilution (4,000 copies) were used in three separate reactions to produce three *gag*, *pol* and 3′ half amplicons (a total of nine reactions). The amplicons were generated in a similar fashion as described above. PCR Amplicons were pooled with their corresponding replicates and run together on one plate along with several other samples. The plasmid clone was submitted to Sanger sequencing and compared to the results of 454 sequencing. Sequencing reads were processed under the same parameters and procedures as previously described [3].

Sequences generated for each sample were then aligned to the NL4-3 reference sequence using BLAST with the following parameters: match: 1; mismatch: -1; gap existence: 1; gap extension: 2. We performed pairwise alignment by applying the NCBI BLASTN program to measure different types of errors (insertions, deletions and substitutions). Second, a perl script was used to parse the BLAST pairwise alignment output XML file. For each pairwise alignment between read and the reference, the script counts the number of aligned nucleotides in the reference sequence, as well as the numbers of insertions, deletions and substitutions in the read compared to the reference. By processing all the reads that aligned to the reference, the total numbers of aligned nucleotides in the reference sequence and different types of errors were calculated. All site-specific errors were then tabulated and the mean frequencies and 95% confidence intervals were calculated.

From these data, we estimated that the average overall process error rate comprising insertions, deletions and mismatches from 454 deep sequencing after data cleaning accounted for 0.19%. We also investigated the error rate in PCR amplification using a NL4-3 plasmid that underwent our three overlapping PCR amplification protocol. As expected the overall error rate averaged across three independent runs increased to 0.32% (*P >* 0.05, Mann Whitney Test). All detected variants found within the plasmid control were only observed at low frequencies (**S6A Fig)** and even when compared between different PCR reactions the maximum frequency that variants were detected at was less than 12% (**S6B Fig)**. Thus, although PCR does increase the mismatch error the rate at which this occurs is unlikely to have influenced our results. In addition, we adopted a stringent cut-off of 1% for variant detection that was three to five times above the calculated error rate, thereby discarding all variability at lower read frequencies.

**Single Genome Amplification and Sequencing of 3′ half genome**

Reverse transcription of RNA was performed with SuperScript III reverse transcriptase (SSIII-RT) following the manufacturer’s recommendation (Invitrogen Life Technologies). Five µl of template was added to 2.5 µM oligoT20 and 0.5 mM of each deoxynucleoside triphosphate in the presence of water to get a final volume of 10 µl. The sample was then heated at 65**°**C for 5 minutes and then the temperature was reduced to 45**°**C for 5 minutes. The following was then added to each cDNA synthesis: 2X First-Strand Buffer, 5 mM DTT, 2 units/µl of RNaseOUT (RNase inhibitor), and 10 units/µl of SSIII-RT. The mixture was then heated at 45**°**C for 90 minutes. After that incubation, 10 units/µl of SSIII-RT was added to each reaction and returned to 45**°**C for 90 minutes. The temperature was increased to 70**°**C for 15 minutes and then each reaction was treated with RNase H at 37**°**C for 20 minutes.

cDNA was serially diluted and amplified in replicate in 96-well plates to identify the dilution yielding PCR success rates of <30% at which point the majority of amplicons are derived from a single copy template based of a Poisson distribution [5] . PCR was performed with 10X Takara Buffer (TaKaRa), 0.6 µM of each primer, 0.25 mM of each deoxynucleoside triphosphate, and 1.25 units/µl of TaKaRa Ex Taq HS DNA polymerase (TaKaRa). FB6 was the sense primer and FB12 was the antisense primer for first round PCR [6]. A second round nested PCR was performed following the same reaction mix but with FB7, the sense primer, and FB13, the antisense primer. The PCR conditions for both reactions were as follows: an initial denaturation step of 94**°**C for 2 minutes; 40 cycles of a denaturation step of 94**°**C for 30 seconds, an annealing step of 60**°**C for 30 seconds, and an extension step of 72**°**C for 5 minutes; and a final extension step of 68**°**C for 10 minutes. The amplified products were sequenced using a GS Junior 454 machine and subjected to further analysis to confirm PCR amplifications from a single template. Each dataset was examined for its homogeneity, abundance of variants exhibiting diversity and lastly compared to results previously obtained from bulk 454 sequencing. This process involves the removal of PCR-generated recombination events, Taq polymerase errors or multiple variant templates. The SGA sequence read data from this study are available at the NCBI under accession numbers KR868816 - KR868946.

**SGA/S Sequence Analysis**

Analysis with Hypermut 2.0 (www.hiv.lanl.gov) was performed to remove sequences that showed enrichment of APOBEC3G/F signatures. Sequences that were determined to have a p-value of 0.05 or lower were considered significantly hypermutated and were excluded from subsequent analysis. Sequence alignments were produced using MUSCLE [7] and were manually edited to optimize codon alignment. A neighbor-joining phylogenetic tree including sequences from all subjects indicated that the sequences from each subject were monophyletic and clustered with strong bootstrap support (data not shown). For each subject, a maximum likelihood phylogenetic tree was inferred using PhyML, version 3.0 [8]. Recombination was assessed by statistical analyses with GARD [9], RDP [10] or Recco [11] recombination identification tools. SGA sequences were visually inspected by Highlighter plots using the Highlighter tool ([http://www.hiv.lanl.gov](http://www.hiv.lanl.gov/)).

# References for Supporting Information

1. Fiebig EW, Wright DJ, Rawal BD, Garrett PE, Schumacher RT, Peddada L, et al. Dynamics of HIV viremia and antibody seroconversion in plasma donors: implications for diagnosis and staging of primary HIV infection. AIDS. 2003;17(13):1871-9. Epub 2003/09/10. doi: 10.1097/01.aids.0000076308.76477.b8. PubMed PMID: 12960819.

2. Yang X, Charlebois P, Gnerre S, Coole MG, Lennon NJ, Levin JZ, et al. De novo assembly of highly diverse viral populations. BMC Genomics. 2012;13:475. Epub 2012/09/15. doi: 1471-2164-13-475 [pii]

10.1186/1471-2164-13-475. PubMed PMID: 22974120.

3. Henn MR, Boutwell CL, Charlebois P, Lennon NJ, Power KA, Macalalad AR, et al. Whole genome deep sequencing of HIV-1 reveals the impact of early minor variants upon immune recognition during acute infection. PLoS Pathog. 2012;8(3):e1002529. Epub 2012/03/14. doi: 10.1371/journal.ppat.1002529

PPATHOGENS-D-11-02202 [pii]. PubMed PMID: 22412369.

4. Macalalad AR, Zody MC, Charlebois P, Lennon NJ, Newman RM, Malboeuf CM, et al. Highly sensitive and specific detection of rare variants in mixed viral populations from massively parallel sequence data. PLoS Comput Biol. 2012;8(3):e1002417. Epub 2012/03/23. doi: 10.1371/journal.pcbi.1002417

PCOMPBIOL-D-11-01679 [pii]. PubMed PMID: 22438797.

5. Rodrigo AG, Goracke PC, Rowhanian K, Mullins JI. Quantitation of target molecules from polymerase chain reaction-based limiting dilution assays. AIDS Res Hum Retroviruses. 1997;13(9):737-42. Epub 1997/06/10. PubMed PMID: 9171217.

6. Miura T, Brockman MA, Brumme CJ, Brumme ZL, Carlson JM, Pereyra F, et al. Genetic characterization of human immunodeficiency virus type 1 in elite controllers: lack of gross genetic defects or common amino acid changes. J Virol. 2008;82(17):8422-30. Epub 2008/06/20. doi: JVI.00535-08 [pii]

10.1128/JVI.00535-08. PubMed PMID: 18562530.

7. Edgar RC. MUSCLE: multiple sequence alignment with high accuracy and high throughput. Nucleic Acids Res. 2004;32(5):1792-7. Epub 2004/03/23. doi: 10.1093/nar/gkh340

32/5/1792 [pii]. PubMed PMID: 15034147.

8. Guindon S, Dufayard JF, Lefort V, Anisimova M, Hordijk W, Gascuel O. New algorithms and methods to estimate maximum-likelihood phylogenies: assessing the performance of PhyML 3.0. Syst Biol. 2010;59(3):307-21. Epub 2010/06/09. doi: syq010 [pii]

10.1093/sysbio/syq010. PubMed PMID: 20525638.

9. Kosakovsky Pond SL, Posada D, Gravenor MB, Woelk CH, Frost SD. GARD: a genetic algorithm for recombination detection. Bioinformatics. 2006;22(24):3096-8. Epub 2006/11/18. doi: btl474 [pii]

10.1093/bioinformatics/btl474. PubMed PMID: 17110367.

10. Martin D, Rybicki E. RDP: detection of recombination amongst aligned sequences. Bioinformatics. 2000;16(6):562-3. Epub 2000/09/12. PubMed PMID: 10980155.

11. Maydt J, Lengauer T. Recco: recombination analysis using cost optimization. Bioinformatics. 2006;22(9):1064-71. Epub 2006/02/21. doi: btl057 [pii]

10.1093/bioinformatics/btl057. PubMed PMID: 16488909.
