# Supplementary figures and images for: Differences in the Selection Bottleneck between Modes of Sexual Transmission Influence the Genetic Composition of the HIV-1 Founder Virus
Source: PLoS Pathog. 2016 May 10;12(5):e1005619. doi: 10.1371/journal.ppat.1005619 (PMC4862634; doi:10.1371/journal.ppat.1005619)

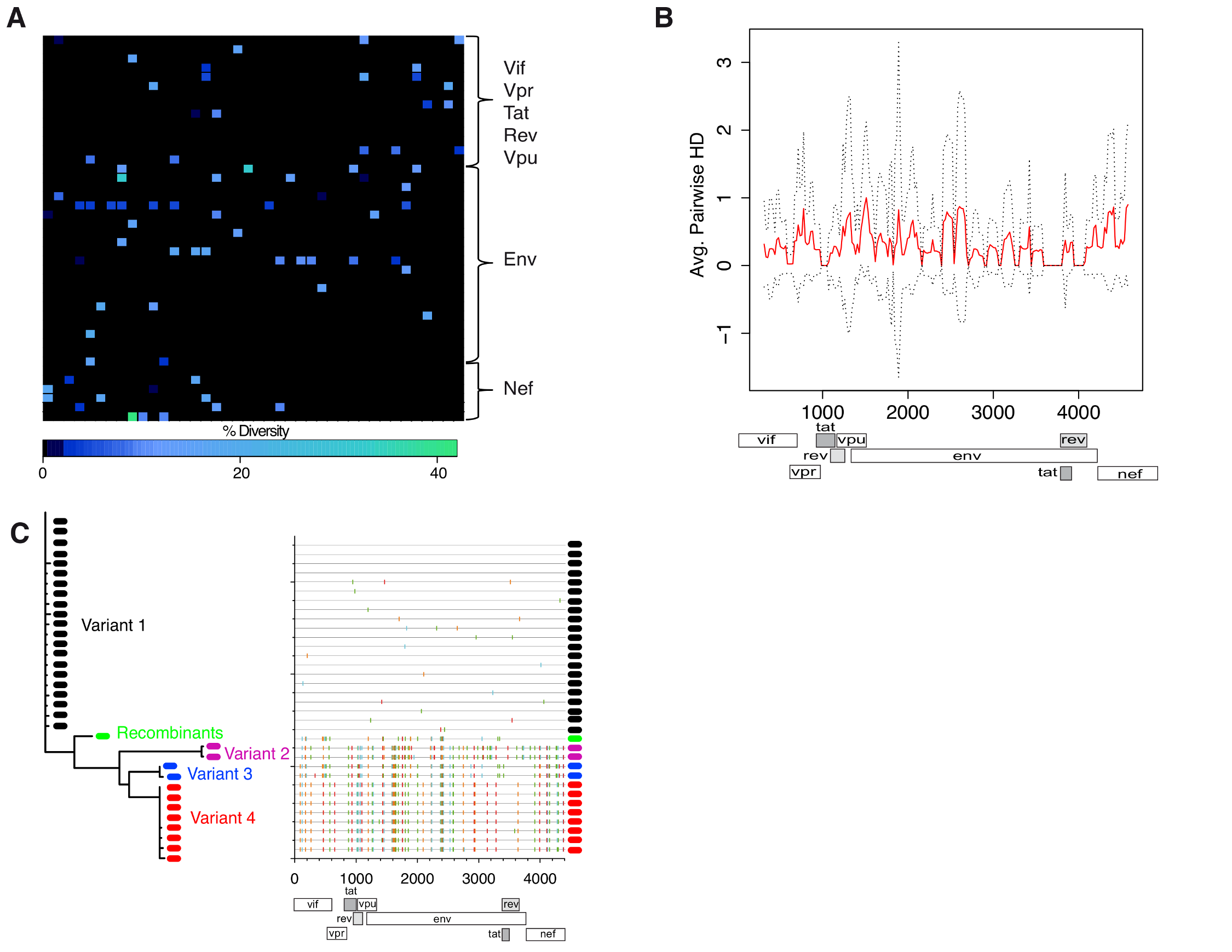

Supplement: S1 Fig — (A) Heatmap from the 454 sequencing data illustrating a diverse number of sites throughout the 3′ half of the genome showing up to 40% codon diversity. (B) The APHD plots showing a mean APHD of 0.306 (red line) and standard deviation (dotted black line) demonstrating a high level of diversity across the 3′ half of the HIV-1 genome. (C) SGA sequences displaying a phylogeny (left) revealing infection by at least four viruses with inter-lineage recombinants. Founder virus lineages are color-coded while recombinant sequences are shown by green symbols. Highlighter plots (right) compare sequences for each subject’s sequence set to an intrasubject consensus (uppermost sequence) and depict the pattern of nucleotide base mutations. Subject 882283 was viral RNA positive but Western blot negative (Fiebig stage II/III of infection). (TIF) [file ppat.1005619.s002.tif]

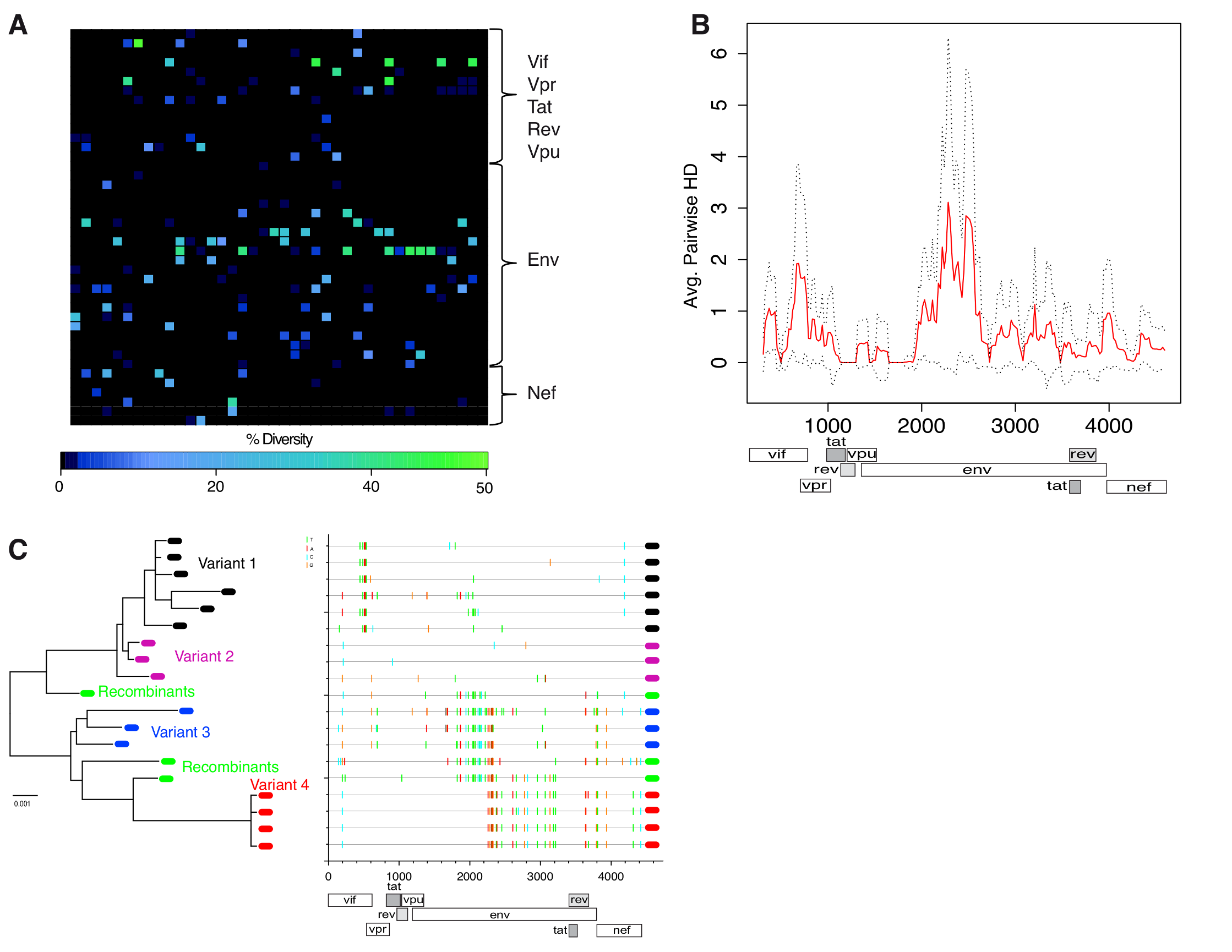

Supplement: S2 Fig — (A) Heatmap from the 454 sequencing data illustrating a diverse number of sites throughout the 3′ half of the genome showing up to 40% codon diversity. (B) The APHD plots showing a mean APHD of 0.593 (red line) and standard deviation (dotted black line) demonstrating a high level of diversity across the 3′ half of the HIV-1 genome. (C) SGA sequences displaying a phylogeny (left) revealing infection by at least four viruses with inter-lineage recombinants. Founder virus lineages are color-coded while recombinant sequences are shown by green symbols. Highlighter plots (right) compare sequences for each subject’s sequence set to an intrasubject consensus (uppermost sequence) and depict the pattern of nucleotide base mutations. Subject 702865 was viral RNA positive but Western blot indeterminate (Fiebig stage IV of infection). (TIF) [file ppat.1005619.s003.tif]

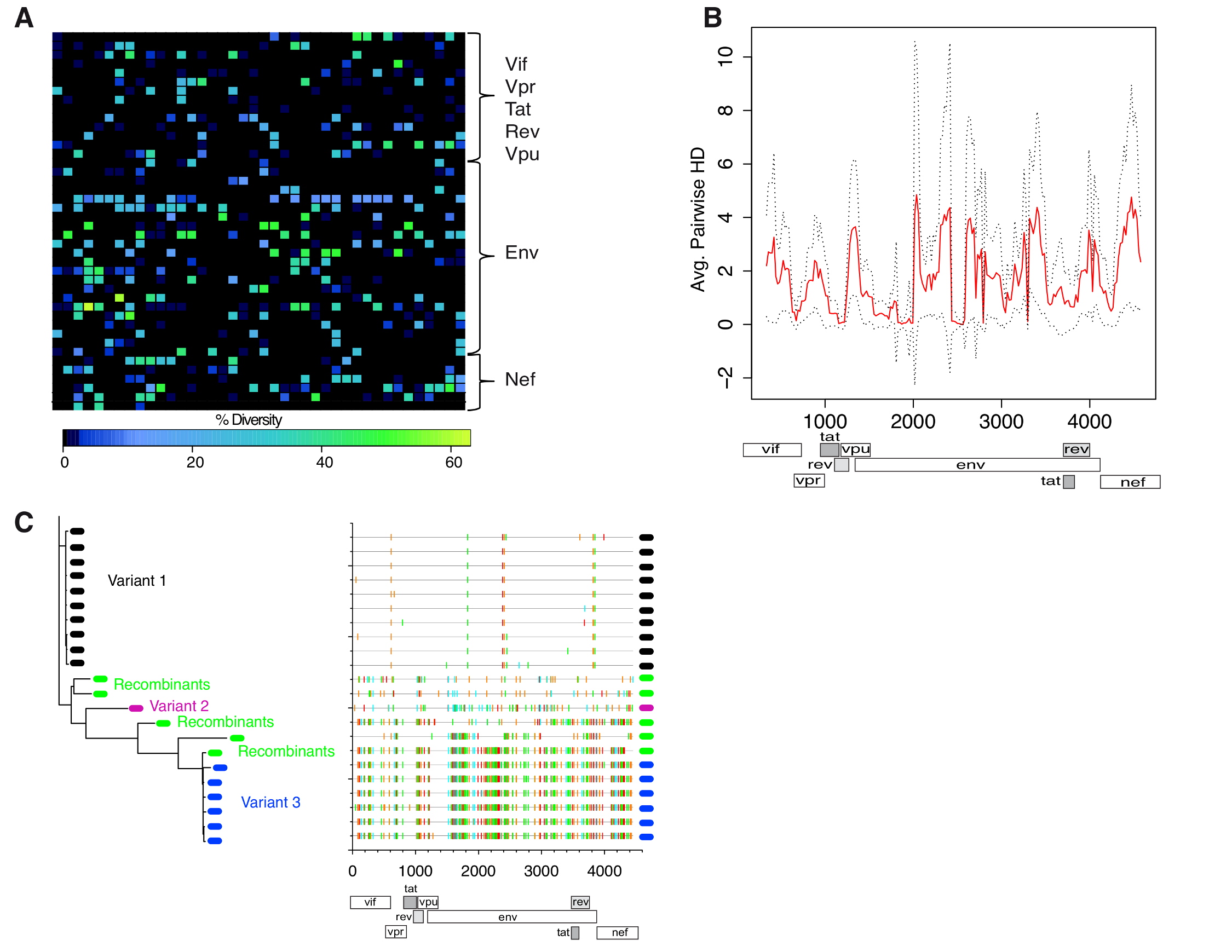

Supplement: S3 Fig — (A) Heatmap from the 454 sequencing data illustrating a diverse number of sites throughout the 3′ half of the genome showing up to 40% codon diversity. (B) The APHD plots showing a mean APHD of 1.718 (red line) and standard deviation (dotted black line) demonstrating a high level of diversity across the 3′ half of the HIV-1 genome. (C) SGA sequences displaying a phylogeny (left) revealing infection by at least four viruses with inter-lineage recombinants. Founder virus lineages are color-coded while recombinant sequences are shown by green symbols. Highlighter plots (right) compare sequences for each subject’s sequence set to an intrasubject consensus (uppermost sequence) and depict the pattern of nucleotide base mutations. Subject 574194 was viral RNA positive but Western blot positive with 3 bands (Fiebig stage V of infection). (TIF) [file ppat.1005619.s004.tif]

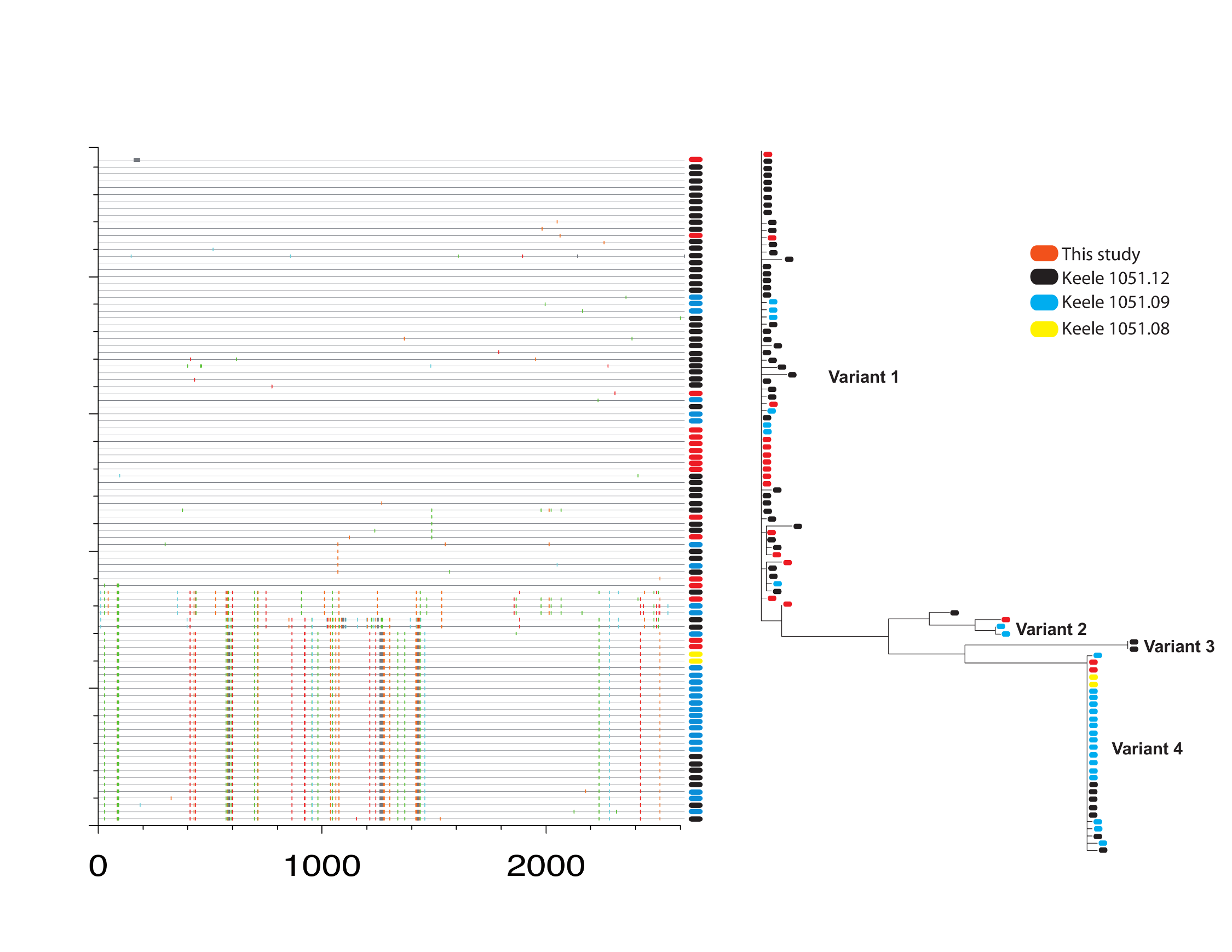

Supplement: S4 Fig — SGA sequences derived from this study (red) were compared to sequences derived from Keele et al. [14] in which 3 additional timepoints were sequenced. Previous analyses by Keele revealed infection by at least 4 founder viruses while in this study we found infection by at least 3 viruses [14]. (TIF) [file ppat.1005619.s005.tif]

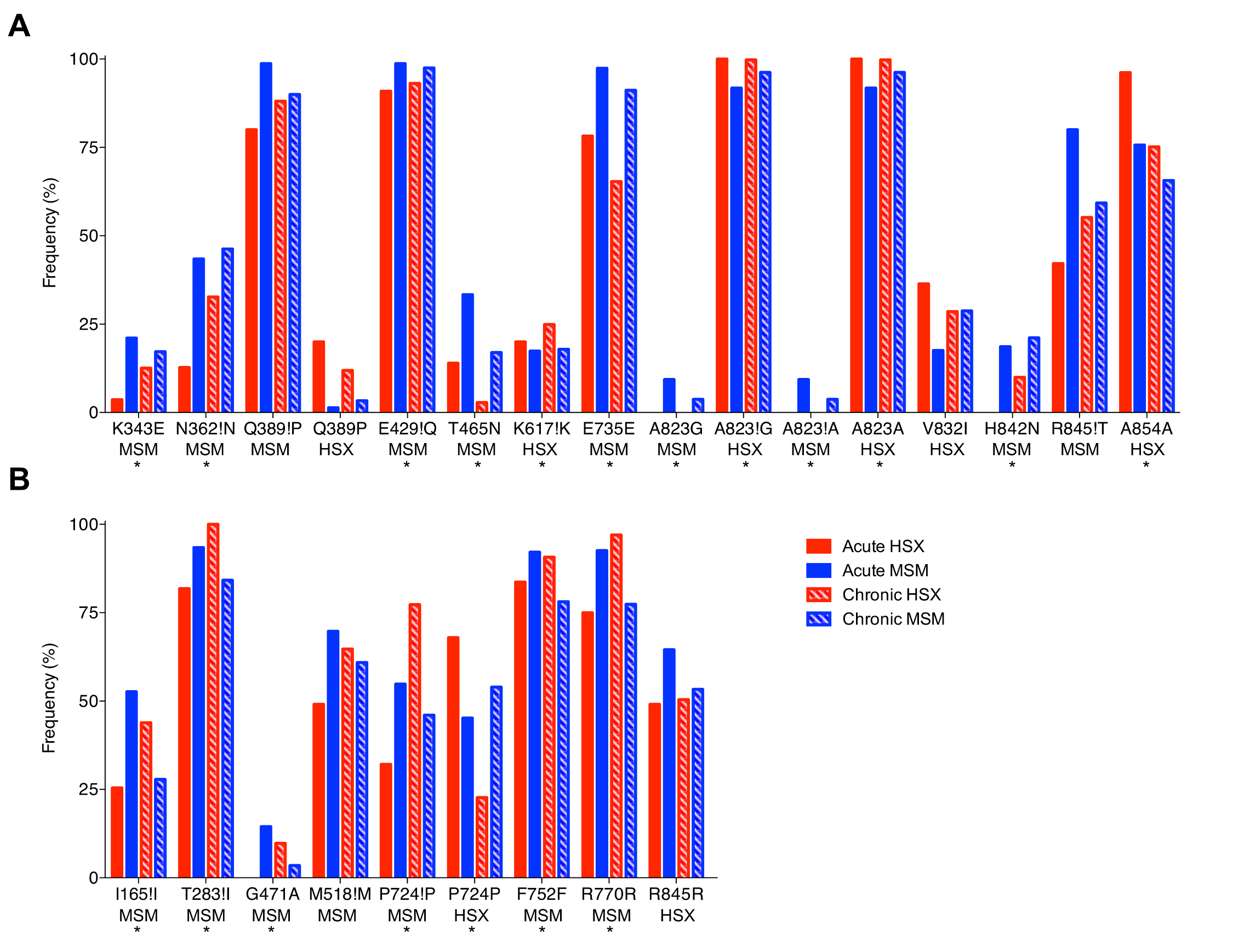

Supplement: S5 Fig — Frequency comparison of previously found signatures sites in our panel of HSX and MSM founder viruses compared to a dataset of chronic HSX and MSM viruses. Chronic viruses comprised 462 SGA/S sequences derived from 24 chronically infected subjects who reported heterosexual as their risk factor for HIV-1 infection and 867 SGA/S sequences from 35 chronically infected MSM subjects. Sites and amino acids examined are listed on the x-axis with the frequency of that amino acid at that position shown on the y-axis. Acute HSX (red bars), acute MSM (blue bars), chronic HSX (red striped bars) and chronic MSM (blue striped bars). (A) Sites that show the same frequency trend in chronic and acute infection are depicted. (B) Sites that show the opposite trend in chronic and acute infection are shown. Sites that showed a statistically significant difference between the chronic stage of infection at a P value of less than 0.05 (Chi-Square test) are indicated by a single asterisk (*). (TIF) [file ppat.1005619.s006.tif]

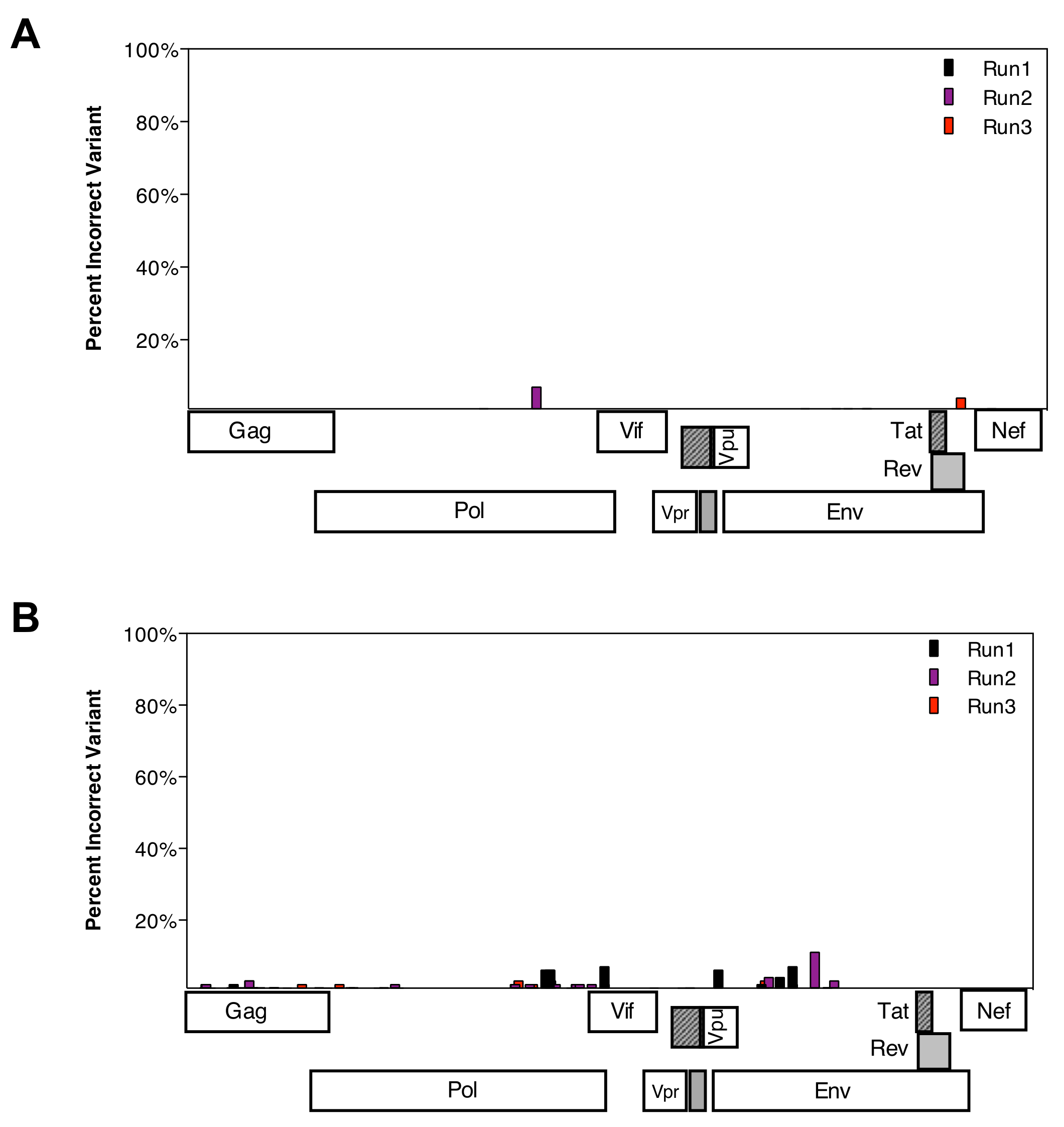

Supplement: S6 Fig — (A) Distribution of point mutation errors across the HIV-1 genome from 3 independent sequencing runs of an HIV-1 NL4-3 plasmid control. (B) Effect of PCR amplification on point mutation variant mismatches across three independent PCR and sequencing runs. (TIF) [file ppat.1005619.s007.tif]
